# Physiologically Based Dissolution Testing in a Drug Development Process—a Case Study of a Successful Application in a Bioequivalence Study of Trazodone ER Formulations Under Fed Conditions

**DOI:** 10.1208/s12249-020-01662-8

**Published:** 2020-06-02

**Authors:** Dorota Danielak, Bartłomiej Milanowski, Krzysztof Wentowski, Maria Nogowska, Michał Kątny, Piotr Rogowski, Łukasz Konwicki, Ewa Puk, Jarosław Pieczuro, Marek Bawiec, Grzegorz Garbacz, Janina Lulek

**Affiliations:** 1 Department of Physical Pharmacy and Pharmacokinetics, Faculty of Pharmacy, Poznan University of Medical Sciences, 6 Święcickiego st, 60-781 Poznań, Poland; 2 Department of Pharmaceutical Technology, Faculty of Pharmacy, Poznan University of Medical Sciences, 6 Grunwaldzka st, 60-780 Poznań, Poland; 3 Biofarm Sp. z o.o, 13 Wałbrzyska st, 60-198 Poznań, Poland; 4 Institute of Computer Engineering, Control and Robotics, Wroclaw University of Technology, 27 Wybrzeże Wyspańskiego st, 50-370 Wrocław, Poland; 5 Physiolution GmbH, Walther-Rathenau Strasse 49a, 17489 Greifswald, Germany

**Keywords:** Trazodone, Generic, Bioequivalence, Extended release, Biorelevant dissolution

## Abstract

Development of generic extended-release (ER) formulations is challenging. Especially under fed conditions, the risk of failure in bioequivalence trials is high because of long gastric residence times and susceptibility to food effects. We describe the development of a generic trazodone ER formulation that was aided with a biorelevant dissolution evaluation. Trazodone hydrochloride 300-mg monolithic matrix tablets were dissolved both in USP and EMA compliant conditions and in the StressTest device that simulated both physicochemical and mechanical conditions of the gastrointestinal passage. The final formulation was tested against the originator, Trittico XR 300 mg, in a randomized cross-over bioequivalence trial with 44 healthy volunteers, in agreement with EMA guidelines. Initially developed formulations dissolved trazodone similarly to the originator under standard conditions (f_2_ factor above 50), but their dissolution kinetics differed significantly in the biorelevant tests. The formulation was optimized by the addition of low-viscosity hypromellose and mannitol. The final formulation was approved for the bioequivalence trial. Calculated C_max_ were 1.92 ± 0.77 and 1.92 ± 0.63 [μg/mL], AUC_0-t_ were 27.46 ± 8.39 and 29.96 ± 9.09 [μg∙h/mL], and AUC_0-∞_ were 28.22 ± 8.91 and 30.82 ± 9.41 [μg∙h/mL] for the originator and test formulations, respectively. The 90% confidence intervals of all primary pharmacokinetic parameters fell within the 80–125% range. In summary, biorelevant dissolution tests supported successful development of a generic trazodone ER formulation pharmaceutically equivalent with the originator under fed conditions. Employment of biorelevant dissolution tests may decrease the risk of failure in bioequivalence trials of ER formulations.

**Electronic supplementary material:**

The online version of this article (10.1208/s12249-020-01662-8) contains supplementary material, which is available to authorized users.

## INTRODUCTION

The global pharmaceutical market requires high-quality generic drugs. Solely in the USA, approximately 90% of prescribed drugs are generic; at the same time, they account for less than a quarter of total expenses on prescription drugs (1). In 2018, savings from generic drug prescription amounted to 292.6 billion dollars in the USA alone (2). Pharmaceutical companies are required to prove bioequivalence of the manufactured generic drug with a brand name product unless the regulatory agency approves biowaiver. This process is time- and cost-consuming. Therefore, the sponsor of the study should make all the efforts to develop a formulation that will ensure the success of the pharmacokinetic bioequivalence trial. Drug pharmacokinetics differs both within and between subjects due to physiological conditions such as sex, age, or genetic polymorphisms of enzymes involved in the metabolism of xenobiotics. Therefore, variability of drug dissolution should be as low as possible. Also, drug release from generic formulation should resemble the brand name product as closely as possible under fasted and fed conditions, if applicable. It is even more critical in modified (MR) and extended-release (ER) formulations. As the ER forms are designed to release the active ingredient over prolonged time, they are prone to food effects (3). Koziolek *et al*. (3) distinguished three categories of food effects relevant for MR and ER dosage forms: (i) drug-related factors including partition coefficient, stability in different pH values, or absorption rate; (ii) formulation-related factors comprising dose, size, excipients, and drug release profiles; and (iii) physiology-related factors including gastrointestinal motility, specific pH profile, gastric emptying, or food composition and caloric content. Novel test methods allow the *in vitro* evaluation of drug dissolution profiles under physiologically relevant conditions. They allow the use of media simulating the composition of fluids in human gastrointestinal tract, such as simulated gastric fluid (SGF) or simulated intestinal fluid (SIF) (4). Additionally, devices such as Dissolution Stress Test (5) or a Fed Stomach Model (6) can effectively simulate shear stresses generated by peristaltics during gastrointestinal passage. As shown in the SmartPill® studies, these contractions generate pressure up to 500 mbar, especially in the antro-pyloric region during gastric emptying (7).

Since the drug delivery of ER products is often dependent on their geometry, the simulation of mechanical stresses in dissolution tests, such as simulated motility forces or dynamic events of transport, can effectively point dissimilarities between ER formulations under biorelevant conditions (8). Different susceptibility of formulations to mechanical stress *in vivo* can cause irregular drug release and increase pharmacokinetic variability (9,10). Therefore, advanced dissolution stress devices can aid the successful development of high-quality generic drugs.

In this paper, we demonstrate the development of a generic trazodone ER formulation. Trazodone is a weak base. This Biopharmaceutics Classification System (BCS) Class II drug is sparingly soluble in water, and its experimentally evaluated logP is 2.9 (11). It is also pH sensitive with a pK_a_ of about 6.74 (12). Trazodone is most commonly used as a salt of hydrochloric acid.

The novelty of the study is an intensive use of bio-predictive, physiology-mimicking dissolution tests during the formulation development process, leading to a successful bioequivalence trial under fed conditions. Until now, the usefulness of the StressTest device was used for the development of novel, pressure-sensitive dosage forms (13) or for an explanation of observed fluctuations in the pharmacokinetics of drugs administered as ER formulations (10). Therefore, the paper presents a new application of the device for bioequivalent generic drug development.

## MATERIALS AND METHODS

### Reagents

#### Trazodone Formulations

Generic trazodone hydrochloride ER tablets (Trazodon XR 300 mg), containing 300 mg of the active ingredient (equivalent to 273.2 mg of trazodone), were manufactured by a direct compression method. The dissolution profiles of the active substance were tested along with the brand name product—Trittico XR 300 mg (Aziende Chimiche Riunite Angelini Francesco A.C.R.A.F. S.p.A.). Six batches, labeled A to F, were tested in biorelevant conditions. The qualitative composition of each analyzed batch is available in Table I. The amount of the active ingredient—trazodone—did not exceed 35% of total tablet mass.

**Table I:** Composition of Developed Formulations Tested in Biorelevant Dissolution Tests. For All of the Ingredients Besides Trazodone Hydrochloride, the Values Are Presented as Mass Percentages of the Total Mass of the Tablet

Ingredient	A	B	C	D	E	F
Trazodone hydrochloride (mg)	300					
Hypromellose 100,000 (%)	16–19	21–24	21–24	5–10	5–10	5–10
Hypromellose 4000 (%)	–	–	–	5–10	5–10	5–10
Microcrystalline cellulose (%)	20–25	20–25	15–20	23–32	20–26	20–26
Silicified microcrystalline cellulose (%)	20–25	15–20	15–20	16–19	16–19	16–19
Mannitol (%)	–	–	3–8	–	3–6	3–6
Talc (%	1–2					
Magnesium stearate (%)	1–2					
Polyvinyl coating	Yes	Yes	Yes	No	No	Yes

#### Dissolution Media

In the development process, both standard and biorelevant media were used. Standard media included 0.1 M hydrochloric acid with 2.92 g/L sodium chloride (pH = 1.2) and 50 mM phosphate buffer pH = 6.0. Biorelevant media were 50 mM phosphate buffer at pH ranging from 4.5 down to 1.8 (with the addition of HCl), and 50 mM phosphate buffer pH = 6.5 with FaSSIF (Fasted State Simulated Intestinal Fluid)/FeSSIF (Fed State Simulated Intestinal Fluid)/FaSSGF (Fasted State Simulated Gastric Fluid) powder (Biorelevant.com Ltd., London, UK) in a concentration corresponding to a fed state. All of the reagents were of analytical grade.

### Dissolution Tests

Each tablet batch was preliminarily tested in a standard dissolution media. Then, it was investigated further in biorelevant conditions resembling fed state. In all biorelevant tests, the dissolution profiles of trazodone from generic formulations were compared with the originator. If the dissolution profile differed significantly from the originator, reformulation and further characterization were performed. The batch with the dissolution profile most comparable with the brand name product was qualified for use in the clinical bioequivalence trial.

#### Standard Dissolution Tests

Standard dissolution tests were performed in a conventional USP dissolution apparatus 2 (Agilent VK7025 and Agilent 708 DS) with a constant paddle rotation at 150 rpm, according to “Guideline on quality of oral modified release products” issued by European Medicines Agency (EMA) (14). The tablets were placed in USP compliant Japanese basket sinkers. The conditions used for dissolution studies were based on a Trazodone once daily patent documentation, No. US7829120B2 (12). Initially, the dissolution medium was 0.1 M hydrochloric acid with 2.92 g/L sodium chloride (pH = 1.2). After 1 h, the medium was exchanged to a 50-mM phosphate buffer pH = 6.0. Dissolution profiles were determined for 24 h. Collected samples were assayed with a high-performance liquid chromatography method, validated according to “Validation of Analytical Procedures: Text and Methodology” guidelines developed by the ICH (15). The chromatographic separation was performed on an XBridge C18 column 3.5 μm, 75 × 4.6 mm (Waters, MA, USA), using acetonitrile and trifluoroacetic acid (0.2%) (35:65; v/v) as a mobile phase, at the isocratic flow rate of 1.2 mL/min. The total run time was 2 min, the injection volume was 5.0 μL, and the detection (UV-Vis) wavelength was set at 246 nm. The column temperature was maintained at 25°C. Additionally, an f_2_ similarity factor was calculated, according to the EMA guideline (16). The f_2_ values greater than 50 ensured the equivalence of both test and originator products in standard dissolution studies.

#### Biorelevant Dissolution Tests

##### Stress Test Device

The dissolution stress test device was first introduced by G. Garbacz and W. Wetschies (9). The core principle of the StressTest device is to simulate the mechanical agitation of physiological intensity that acts on a solid dosage form during the GI transit as well as the physicochemical conditions in the subsequent sections of the GI. A detailed description of the device is given elsewhere (5).

##### Test Setup Parameters

**Fig. 1:**
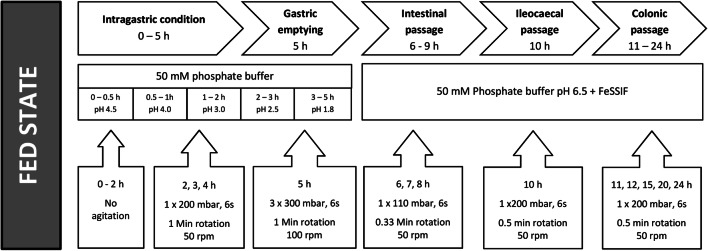
Setup of the program for biorelevant dissolution tests

Dissolution tests were designed to simulate the fed intake conditions of the bioequivalence trial. First, the medium was 1100 mL 50 mM phosphate buffer at pH 4.5. The pH of the medium was gradually decreased to 1.8 by addition of 5 M hydrochloric acid; then, after 5 h the pH was adjusted to pH 6.5 by the addition of 40% sodium hydroxide, and 40 mL of a FaSSIF/FeSSIF/FaSSGF concentrate solution in a 50 mM phosphate buffer concentrate was added (77.9 g of the powder in a 50 mM phosphate buffer, filled up to a final volume of 240 mL, stirred until dissolved, and allowed to settle for at least 1 h before addition to the media). The final concentration of the FaSSIF/FeSSIF/FaSSGF in the simulated intestinal fluid was 11.2 g/L, corresponding to the fed state. The media change pattern was aligned with the simulation of the mechanical aspects of the GI tract. The stress program was set up. Figure 1 presents stress as well as the media type and change pattern used in the present biorelevant dissolution studies.

##### Determination of Trazodone in Biorelevant Media

The amount of the drug dissolved was determined every 10 min using online closed-loop UV-Vis spectroscopy. The samples were filtered through a PES (polysulfone) filter with a pore size of 1 μm (Sartorious, Göttingen, Germany) and analyzed in a flow-through mode. Flow-through quartz cuvettes of a 5-mm path length (Hellma, Müllheim, Germany) were used with an Agilent 8543 spectrophotometric system (Agilent, Santa Clara, USA) with the photometers set to a differential mode at two wavelengths of *λ* = 313 nm for trazodone signal and *λ* = 450 nm for the background, respectively.

### Bioequivalence Study

The bioequivalence study was a single-center, single-dose, open-label (laboratory blinded), randomized, four-period, four-way cross-over study, according to the “Guideline on the pharmacokinetic and clinical evaluation of modified release dosage forms” (EMA/CPMP/EWP/280/96 Corr1) (17). The protocol of the study was approved by the Independent Ethics Committee at the Regional Chamber of Physicians in Warsaw, and by the Office for Registration of Medicinal Products, Medical Devices and Biocidal Products in Poland. The trial was registered in the EU Clinical Trial Register under the number 2018-000598-57.

Sustained and predictable release of the drug from the ER dosage is more challenging in fed conditions than in a fasted state, both in dissolution studies and in the proper setup of the clinical study protocol. Therefore, in this paper, we will present the results of the bioequivalence trial performed under fed conditions only.

#### Study Group

The minimum sample size needed to adequately assess the bioequivalence of two trazodone formulations was calculated as suggested by Diletti *et al*. (18). Based on the reported clinical studies NCT00839072 (19) and NCT01121900 (20), the C_max_ of trazodone after administration of 300 mg trazodone hydrochloride varies intra-individually by approximately 29%. Assuming the power of the test at 80% and significance level *α* = 0.05, the bioequivalence assessment required at least 38 subjects.

The study included forty-four healthy subjects of both sexes. The characteristics of the study group are presented in Table II. Each subject signed an Informed Consent Form. Before and during the study, the subjects had to refrain from the use of other drugs, products containing nicotine, alcohol, caffeine, or grapefruit juice. Each subject had the right to withdraw from the study. The participation of a subject might have been discontinued for reasons including adverse events and study protocol deviation.

**Table II:** Characteristics of the Study Group. Data Are Presented as Means ± Standard Deviations

Parameter	Female (*n* = 11)	Male (*n* = 33)
Age (years)	39.3 ± 11.4	33.4 ± 9.3
Weight (kg)	66.0 ± 7.4	74.5 ± 8.3
Body mass index (kg/m^2^)	23.8 ± 2.8	23.4 ± 2.1

#### Protocol

A single dose of the test product (Trazodon XR 300 mg) or the brand name product (Trittico XR 300 mg), both containing 300 mg trazodone hydrochloride, was administered by the oral route. The administration was within 30 min after the start of a standardized high-fat meal, with 250 mL of still, room temperature water. No other fluid intake was allowed from 2 h before until 1-h post-dosing. Additionally, fluids were administered as follows: 250 mL of fluid with breakfast, 150 mL of still water at 1, 2, and 3 h post-administration, 200 mL fluid with meals at 4 and 12 h post-administration, and 100 mL at 5, 6, 7, 8, 9, 10, and 11 h post-administration. Standardized lunch and supper were served at about 4 and 12 h after drug administration.

From each subject 4 mL of full blood was collected before drug administration (time “0”), and at 1, 2, 3, 4, 5, 6, 8, 9, 10, 11, 12, 13, 14, 15, 16, 20, 24, 28, 32, 36, 42, 48, and 72 h after the intake of the drug. The blood samples were taken before the intake of fluids. Full blood was collected through heparinized capillaries into a vacuum blood collection system. Immediately after collection, the samples were centrifuged, and the plasma was transferred into separate tubes, stored in dry ice, and shipped into the laboratory for trazodone determination. Any deviations from the sampling protocol were noted, and the actual sampling times were reported for further analysis.

#### Determination of Trazodone Concentrations and Pharmacokinetic Parameter Calculations

Plasma samples were shipped in dry ice to an external, certified laboratory for the determination of trazodone. The drug concentrations were determined with a high-performance liquid chromatography mass spectrometry method (LC-MS), in a multiple reaction monitoring mode (MRM). The method was validated according to the EMA guidelines in terms of selectivity, accuracy, and precision (21). The lower limit of trazodone quantitation was 10 ng/mL.

The pharmacokinetic evaluation was planned in agreement with the guideline CPMP/EWP/QWP/1401/98 Rev. 1/Corr** (16) and EMA/CPMP/EWP/280/96 Corr1 (17). Following pharmacokinetic parameters were considered for bioequivalence evaluation: area under the time-concentration curve from time 0 to 72 h (AUC_0-t_), area under time-concentration curve extrapolated to infinity (AUC_0-∞_), and maximum concentration obtained directly from the measured concentrations (C_max_). Additional secondary parameters included time to C_max_ (T_max_) and plasma half-life (T_1/2_) that was calculated from 0.693/K_el_, where K_el_ represents the elimination rate constant determined through a linear regression. Of note, only the actual sampling times were used. The parameters were calculated by the linear-log trapezoidal rule in Phoenix WinNonlin software, build 8.1.0.3530 (Certara USA, Princeton, NJ, USA).

Bioequivalence after a single dose of trazodone 300 mg under fed conditions was established upon a ratio of test and reference product parameters. Before the analysis, the parameters were log-transformed. The 90% confidence interval for the ratio should be contained within 80.00–125.00%. The pharmacokinetic parameters were analyzed with ANOVA test, with factors for sequence, subject within sequence, period, and treatment. Also, the Schuirmann’s two one-sided parametric T tests were calculated with the null hypothesis of bioinequivalence at the 5% (*α* = 0.05) level of significance.

## RESULTS

### Standard Dissolution Tests

**Fig. 2:**
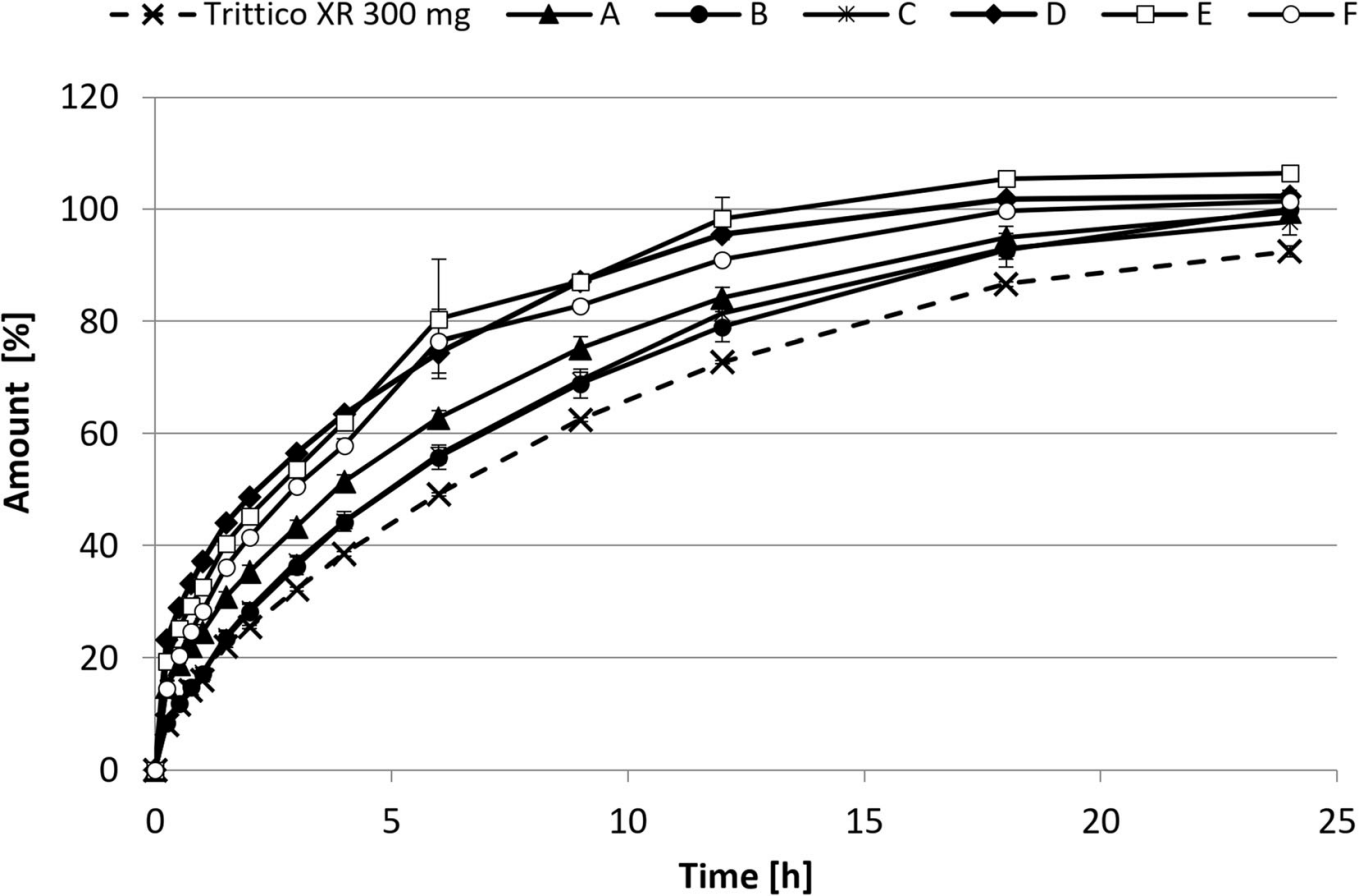
Trittico XR 300 mg and Trazodon XR 300 mg dissolution profiles obtained in standard dissolution tests, according to USP. Data are presented as means (*n* = 4 for Trittico XR 300 mg, A–D, *n* = 2 for E–F) with standard deviations as whiskers

The release profiles of trazodone from tested formulations are presented in Fig. 2. All of the tablets release the active substance steadily over 24 h. Most of the formulations released less than 30% of trazodone within the first hour, no less than 70% after 12 h, and at least 80% after 24 h from the beginning of the dissolution study. The originator released trazodone slower than any of the tested formulations. Batches A, B, and C resembled most the release profile of the originator. In contrast, batches D, and E released the active ingredient at a noticeably faster rate. This observation also holds for the final batch F that was the model for the clinical batch. The calculated f_2_ factors were as follows: batch A = 51.4, batch B = 72.3, batch C = 69.0, batch D = 32.9, batch E = 33.8, batch F = 44.3.

### Dissolution Tests in Biorelevant Conditions

**Fig. 3:**
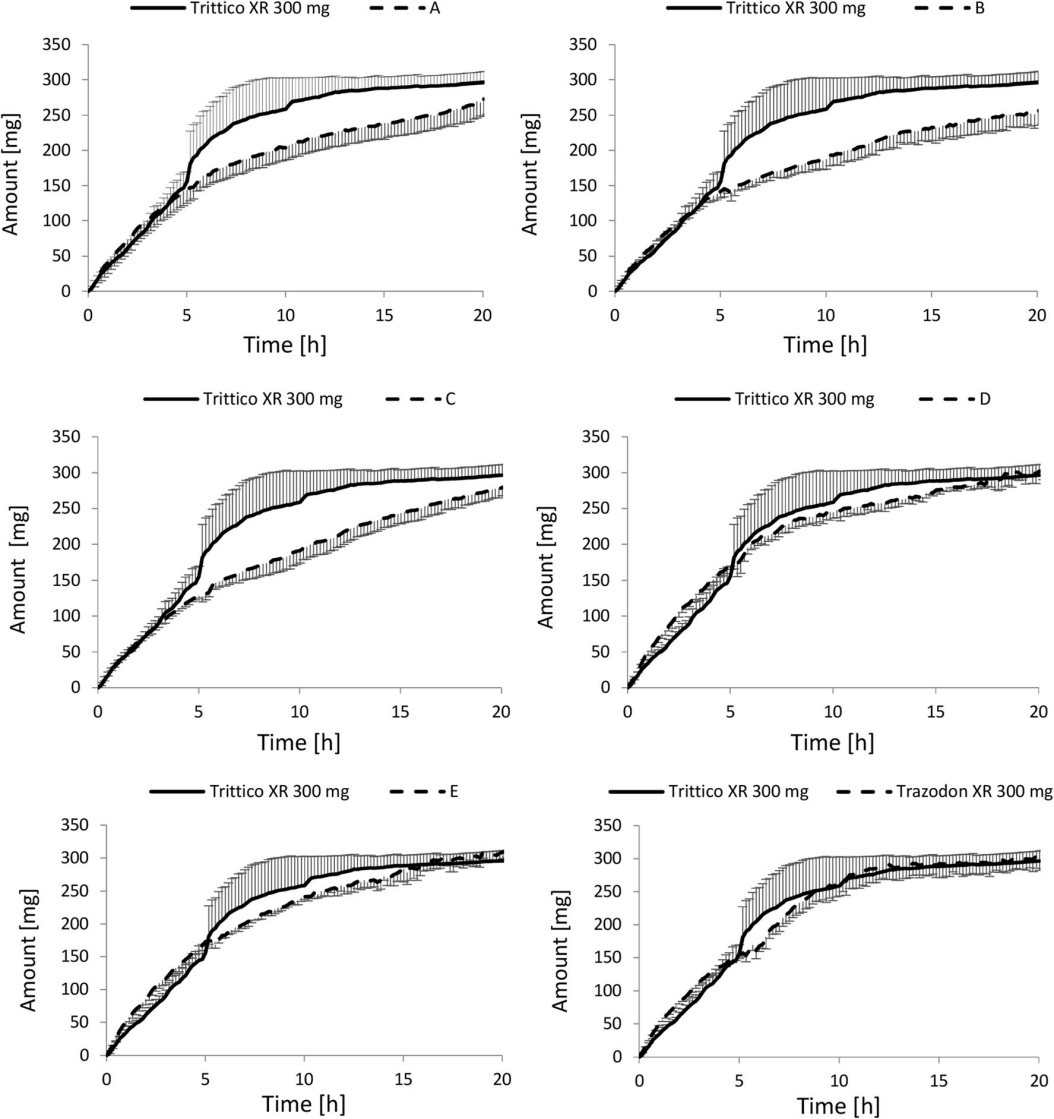
Dissolution profiles of trazodone from tested Trazodon XR 300 mg batches (A–F) *versus* Trittico XR 300 mg obtained under simulated fed conditions. Data are presented as means (*n* = 6) with standard deviations as whiskers

First developed batches—A, B, and C—resembled the trazodone release profile of the originator during the first 5 h (Fig. 3). However, the drug release changed noticeably after the introduction of 300 mbar stress mimicking the gastric emptying and transition from gastric to intestinal media. The mechanical stress affected the dissolution of the originator more than all of the tested batches. Susceptibility of the originator to the events of the mechanical stress of biorelevant fortitude caused a faster release of trazodone and the complete dissolution of tablet matrices. At the same time, the generic matrices resisted biorelevant mechanical agitation and were deformed only slightly. Therefore, further reformulation was required.

Batches D and E were more similar to the brand name product. The final batch F, that was approved for use in the clinical trial, was developed upon batch E. The only difference between batches E and F was a polyvinyl coating that did not influence the release of trazodone from the tablets, as shown in Fig. 3. Batch D was not chosen for further studies because of distinctly different dissolution characteristics under fasted conditions. However, the characterization of drug release under fasted conditions is outside of the scope of this paper.

### Clinical Bioequivalence Trial

Thirty-nine subjects completed the study. As stated above, the trial required a minimum of 38 participants to assess bioequivalence with a sufficient power. Table III presents the calculated pharmacokinetic parameters for both formulations

**Table III:** The pharmacokinetic parameters calculated from the results obtained in the bioequivalence study under fed conditions for the brand name product (Trittico XR 300 mg) and the test product (Trazodon XR 300 mg) (*n* = 39). The data are presented as means ± standard deviations

Parameter	C_max_ [μg/mL]	AUC_0-t_ [μg∙h/mL]	AUC_0-∞_ [μg∙h/mL]	T_max_ [h]	T_1/2_ [h]
Trittico XR 300 mg	1.92 ± 0.77	27.46 ± 8.39	28.22 ± 8.91	7.46 ± 2.29	9.71 ± 2.75
Trazodon XR 300 mg	1.92 ± 0.63	29.96 ± 9.09	30.82 ± 9.41	7.69 ± 2.07	9.52 ± 3.72

AUC_0-∞_ - area under the time-concentration curve extrapolated to infinity, AUC_0-t_ - area under the time-concentration curve between time 0 to 72 hours, C_max_ - maximum concentration, T_1/2_ - plasma half-life, T_max_ – time to maximum concentration

**Fig. 4:**
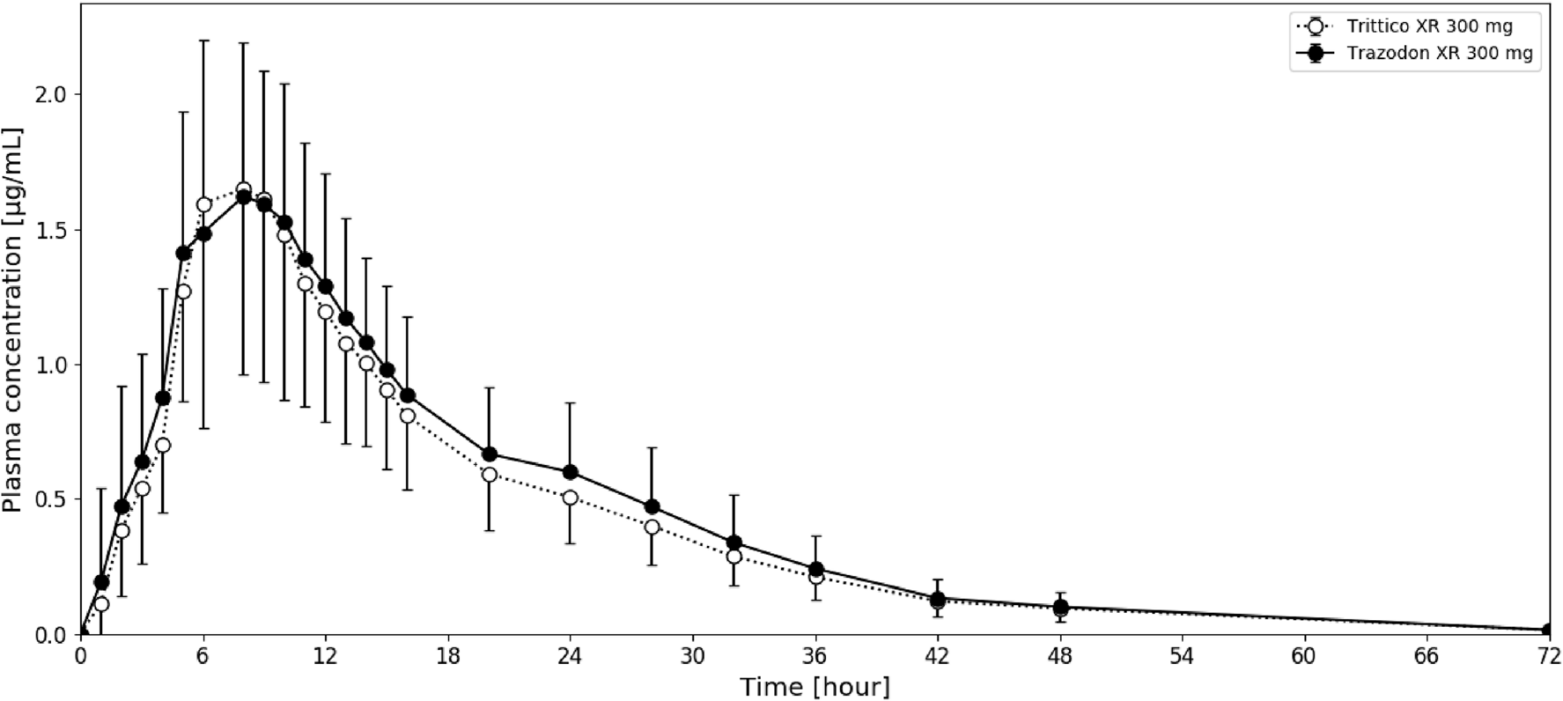
Pharmacokinetic profiles of Trittico XR 300 mg (originator product) *versus* Trazodon XR 300 mg (test product). Data are presented as means with standard deviations as whiskers (*n* = 39)

. Both primary and secondary parameters of the test formulation (Trazodon XR 300 mg) resembled those calculated for the brand name product (Trittico XR 300 mg). Pharmacokinetic profiles of both formulations were similar (Fig. 4). Individual pharmacokinetic profiles are available in the Online Supplementary Data file (Supplement 1). The intra-subject variability for AUC_0-t_, AUC_0-∞_, and C_max_ was 11.56%, 10.74%, and 22.18%, respectively. The inter-subject variability for AUC_0-t_, AUC_0-∞_, and C_max_ was 27.80%, 28.83%, and 28.11%, respectively.

ANOVA test showed that the sequence and period effects were negligible for all primary parameters. One of the statistically significant factors was a joint subject and sequence effect. These parameters affected all three primary parameters (*p* < 0.0001). Also, the type of formulation (test product or the brand name product) influenced the exposure to trazodone, expressed as AUC_0-t_, and AUC_0-∞_ (*p* = 0.0014 and *p* = 0.0005, respectively). For C_max_ this effect was not statistically important (*p* = 0.5955).

The results of the bioequivalence assessment presented in Table IV show that the test product fulfilled the criteria for bioequivalence with the brand name product under fed conditions

**Table IV:** Bioequivalence assessment of the test product (Trazodon XR 300 mg) against the brand name product (Trittico XR 300 mg). The data are presented as a ratio of pharmacokinetic parameters with 90% confidence intervals (CI)

Parameter	Ratio (test/reference)	Lower 90% CI	Schuirmann’s *t*-test *p* value	Upper 90% CI	Schuirmann’s *t*-test *p* value
AUC_0-t_	109.425	104.712	< 0.0001	111.350	< 0.0001
AUC_0-∞_	109.660	105.263	< 0.0001	114.240	< 0.0001
C_max_	102.694	94.444	< 0.0001	111.664	0.0002

AUC_0-∞_ - area under time-concentration curve extrapolated to infinity, AUC_0-t_ - area under the time-concentration curve between time 0 to 72 hours, C_max_ - maximum trazodone concentration

.

## DISCUSSION

In the present study, we demonstrate how the use of biorelevant methods, i.e., the biorelevant stress test device, supported the development of a generic ER trazodone formulation. The test protocols were utilized to predict the drug delivery behavior of the tested formulations. In this context, the performed study served as a proof-of-concept for the predictive power of the StressTest device. As a result, the developed formulation fulfilled the bioequivalence criteria under fed conditions.

The successful formulation of a generic ER dosage form is a complex and challenging process. In general, if the originator and generic formulations are similar, they should also have similar dissolution characteristics under every condition tested. However, the formulation can be considered as equivalent only if the bioequivalence trial is passed under the same test conditions (fasted and/or fed) as the originator. It becomes challenging if the exposure to the drug differs significantly between fasted and fed states. This phenomenon is observed for trazodone. Karhu *et al*. (22) showed that pharmacokinetics of a once-daily Trazodone Contramid 300 mg formulation differed significantly between fasted and fed conditions. The AUC between these two states was similar. However, after a high-fat meal, the C_max_ of trazodone was 86% higher compared with fasted conditions. The C_max_ values falling outside 80–125% confidence intervals show that two products are not equivalent.

Consequently, the fed conditions can be considered as more difficult with respect to the development of generics. Therefore, the present paper aims at the description of the product performance under fed conditions. The strict criteria for primary pharmacokinetic parameters obtained in the bioequivalence trial result in setting up requirements for the pharmaceutical development and characterization of oral medicines in the preclinical stage. Such characterization, especially when performed with biorelevant methodology, may reduce the risk of failure and accelerate the development process.

Adequate reflection of luminal conditions requires more than the compendial media. Food ingredients such as lipids, proteins and their digestion products act as natural surfactants affecting both the solubility of the active pharmaceutical ingredient (API), as well as hydration and erosion processes of MR matrices (7). These components are also present in biorelevant media, such as simulated gastric fluid, fed state simulated intestinal fluid, fasted state simulated intestinal fluid, milk, and nutritional drink. Therefore, these media should be used to predict the disintegration of ER matrices (23,24). The type of food also affects the tablet erosion process. In fed conditions the tablets disintegrate slower in comparison with the fasting state, and thus drug dissolution in the stomach is delayed (25,26). For this reason, we adjusted the study protocol to reflect the physico-chemical properties of gastrointestinal fluids more accurately than conventional dissolution media. The media composition and pH profiles and transit times used in the present study were set according to Koziolek *et al*. (27,28).

The drug delivery behavior of monolithic hydrogel matrix tablets, such as ones developed in this study, may be remarkably affected by the mechanical and hydrodynamical agitation. Motility forces produce such stress during the physiologic events of transport such as gastric emptying, and ileocaecal and colonic passage (29). In some cases, mechanical agitation results in deformation, fast erosion of the tablets matrices, and an increase in the drug delivery rate or even dose dumping (29,30). Such agitation may be more pronounced under fed conditions due to increased motoric activity and passage times through the highly active proximal parts of gastrointestinal tract. Thus, in the present study, we used a test program with differing patterns intended for the simulation of the fed state (Fig. 1). The arrangement of the stress program was derived from the *in vivo* studies performed using telemetric capsules (7,27,31). For the fed state, moderate 200-mbar intragastric stress events were programmed within the first 2–4 h, followed by the major gastric emptying stress (300 mbar) at 5 h. Then, after the transition to ‘intestinal’ conditions, regular stress of 110 and 200 mbar was introduced. In summary, the fed conditions proposed in our study (Fig. 1) reflect these *in vivo* findings according to both composition, residence times, and stress patterns.

All of the tested tablets were monolithic hydrophilic matrix systems. First batches (A–C) consisted mainly of high-viscosity hydroxypropyl methylcellulose (hypromellose, HPMC). In the dissolution study performed according to USP, they released the drug steadily, reaching almost 95% of the dose labeled within 24 h. Subsequent batches, D and E, were characterized by a noticeably faster drug release than batches A–C. In these two batches, the amount of Hypromellose 100000 was decreased to less than 10% of the total tablet mass, and a low viscosity Hypromellose 4000 was introduced; also, batch E included a small amount of mannitol. Final batch F, which differed from batch E only by a presence of polyvinyl coating, had a comparable dissolution profile. Increased release of the drug was also achieved by increasing the amount of microcrystalline cellulose. Properties of hypromellose may explain observed dissolution performance. Of note, all of the batches tested in the USP apparatus had different dissolution kinetics as compared with the originator, Trittico XR 300 mg. As revealed later in the test under biorelevant conditions, the batches with the dissolution profiles that were most similar to the originator performed worse, especially after the transition to media simulating intestinal fluids.

As shown by Conti *et al*. (32,33), high-viscosity polymers release the drug through a diffusion-controlled mechanism, as expressed by Fick’s law, while low viscosity gelling agents promote erosion of the swollen polymer. Poorly water-soluble drugs, such as trazodone under the simulated intestinal conditions, are mostly released through the latter mechanism (34). Additionally, high molecular viscosity polymers may decrease the drug release rate (35). Other properties of hypromellose, such as a higher percentage of hydroxypropoxy groups or particle size, may increase the dissolution rates (36,37). Other excipients used for the preparation of the batches include microcrystalline cellulose and silicified microcrystalline cellulose. They show good binding properties, are compatible with a broad range of drugs, and are physiologically inert; silicified microcrystalline cellulose also has an increased surface area and improved flow characteristics (38,39). Lastly, mannitol can also increase drug release due to the faster uptake of water (40). All of these excipients contributed to an improved dissolution of trazodone from the test tablets.

Interestingly, the dissolution profile of the originator was characterized by an increased release of the active ingredient after transition to intestinal media (Fig. 3). None of the generic products exhibited such a behavior. Trazodone is a weak base with a pK_a_ = 6.74, and it is most commonly used as trazodone hydrochloride. This salt is sparingly soluble in water, but its solubility increases with an increased acidity of the media (41). Thus, in the upper gastrointestinal tract, where pH is below trazodone pK_a_, it dissolves well. Owing to the high volume, weak buffering capacity of the dissolution media, and presence of natural surfactants, the solubility of trazodone is far below the equilibrium solubility. Solubility of trazodone hydrochloride in the simulated intestinal media estimated during the course of our experiment (0.15 mg/mL, data not shown) was much lower than in pure water (18 mg/mL, (41)). Therefore, the higher release of trazodone from Trittico XR 300 mg may result from an extensive stress corresponding to the gastric emptying and it is not an artifact related to the solubility of trazodone. In the case of solubility issues, similar tendencies would also be observed for the generic batches. It underlines a potential advantage of the Stress Test device to discriminate between stress-susceptible and robust extended release dosage forms. In contrast with products developed in the present study, the originator product relies on granulated cross-linked high-amylose starch (Contramide®), mixed with hypromellose, anhydrous colloidal silica, and sodium stearyl fumarate (42). Contramide swells and forms a rubbery gel, similarly to hypromellose (43), but at the same time it is prone to degradation and erosion caused by α-amylase (44). Also, the addition of hypromellose may greatly affect dissolution from cross-linked amylose-based tablets and cause different pharmacokinetic profiles under fasted and fed conditions, as shown by Lenaerts *et al*. (45). The composition of Trittico XR 300 mg may therefore explain observed differences in the trazodone bioavailability under fasted and fed conditions and underlines the importance of biorelevant conditions for a thorough examination of dissolution profiles during product development and release of the clinical batches.

The study concluded with a successful clinical bioequivalence trial under fed conditions. A statistically significant subject-sequence effect can be explained by a relatively high intra-subject variability in the studied population. The type of formulation (the brand name product *vs.* tested product) was shown to be significant for AUC_0-t_ and AUC_0-∞_ in the latin-square ANOVA. However, all of the primary pharmacokinetic parameters fell within the assumed confidence intervals. Therefore, the formulation effect may be considered as negligible, and the two formulations may be concluded as bioequivalent under fed conditions. Besides the development of a formulation with an optimal release profile, the design of the study also contributed to the success in the clinical trial. According to the protocol, not only meals but also fluid intake were tightly scheduled. In the proposed regimen all subjects received a specific liquid volume every hour after administration. A recently established consortium in Understanding Gastrointestinal Absorption-related Processes (UNGAP) investigated the food-drug interactions that may influence the absorption of orally administered drugs (46). According to their state-of-art review, the volume of fluids present in the lumen is one of these factors. First, it influences the concentration-driven passive uptake of the drug and saturable membrane transporters, and second, it exerts an effect on formulation transit times. Another fact is that a standard breakfast is a high-fat energy-rich meal with a high fraction of solids. In stomach it creates a layered, heterogeneous mass that contains layers of solids, fats and fluids (16). Water, a drink of choice in clinical bioequivalence trials, does not mix well with gastric contents rich in fats. Instead, it rapidly follows a so-called stomach road, also known as *Magenstrasse*, and is emptied from the stomach. In case of ER formulations, this may cause even life-threatening consequences due to dose dumping. Also, *ad libitum* intake of water during a controlled clinical trial with ER formulations can lead to a “double peak” phenomenon and contribute to the pharmacokinetic variability (47).

Another aspect is tablet residence time and location in the stomach. If a tablet is taken under fed conditions, it may remain in a fundus region of the stomach; this part of the stomach is poorly mixed and acts as storage (48). If the gastric emptying is delayed, the drug is released slowly and accumulates in the proximal stomach (48). Then, after the gastric emptying, it appears in plasma after a long lag phase and at high concentrations. In consequence it may cause erratic pharmacokinetic profiles and ultimately a failure of the bioequivalence trial. Proposed frequent drinking schedule aimed to reduce the risk of drug accumulation in the fundus and significantly contributed to the success of the trial.

As shown in this study, the development of a pharmaceutically equivalent ER dosage form with a BCS II class active ingredient is a complex process. Simple, compendial methods may not be adequate to ascertain the success in an expensive bioequivalence trial. Also, the use of solely biorelevant media may not be sufficient. In the present study, tested and reference products differed most significantly after the introduction of physiologically relevant stress. Therefore, we confirmed that for monolithic ER formulations the gastrointestinal stress could be an essential element of dosage development. It should be pointed out that all the literature data available so far, describing the usability of the StressTest device, concern only its use for prediction of the dissolution behavior of ER/MR products under fasted conditions. Consequently, the present manuscript represents an original work that describes for the first time the application of the StressTest device and test protocols capable of predicting the *in vivo* drug delivery behavior under the fed state. The obtained results are supported by the outcome of the clinical trial being a part of the study.

## CONCLUSIONS

In summary, the present study shows that a preclinical development of ER formulations may be aided by advanced dissolution studies that take into account not only the composition of luminal fluids and respective residence times but also timing and fortitude of the physiological mechanical stress that occurs during the gastrointestinal passage.

## Electronic supplementary material

ESM 1 (PDF 3410 kb)
