# Supplementary material for: Physiologically Based Dissolution Testing in a Drug Development Process—a Case Study of a Successful Application in a Bioequivalence Study of Trazodone ER Formulations Under Fed Conditions
Source: AAPS PharmSciTech. 2020 Jun 2;21(5):161. doi: 10.1208/s12249-020-01662-8 (PMC7266804; doi:10.1208/s12249-020-01662-8)

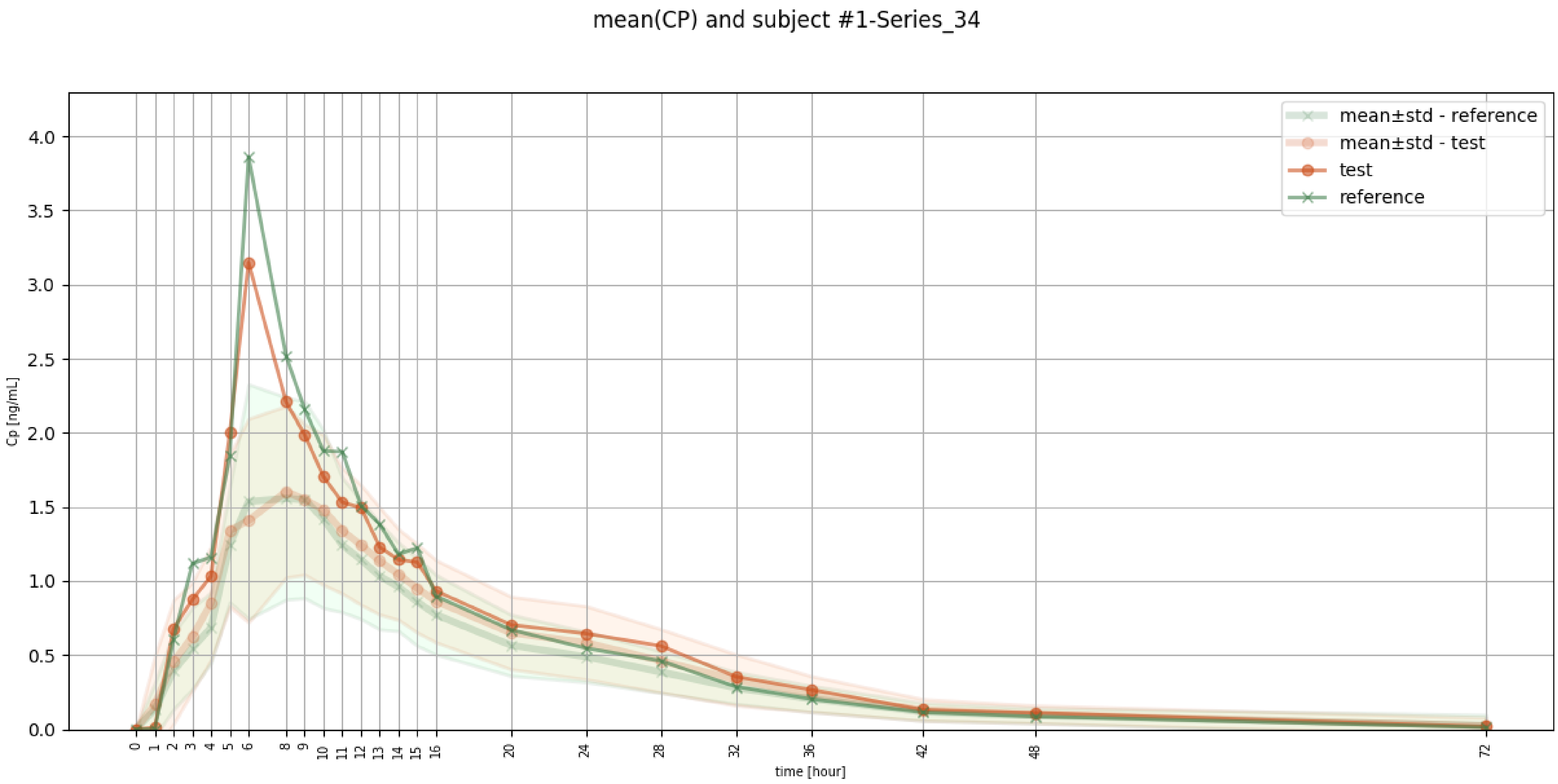

mean(CP) and subject #1-Series_34
mean±std - reference
mean±std - test
test
reference
Cp [ng/mL]
time [hour]
0.0
0.5
1.0
1.5
2.0
2.5
3.0
3.5
4.0
0 1 2 3 4 5 6 8 9 10 11 12 13 14 15 16 20 24 28 32 36 42 48 72

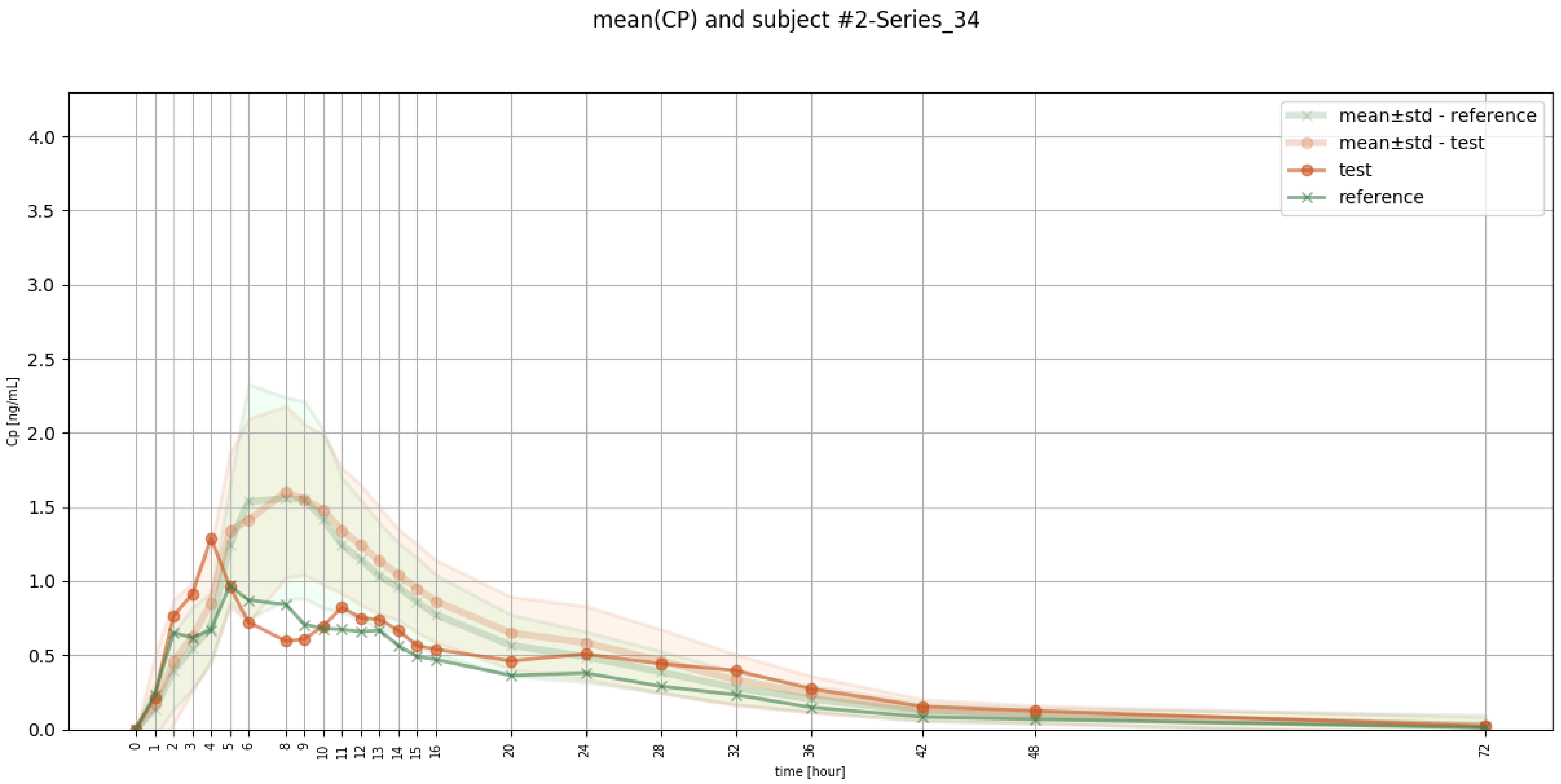
mean(CP) and subject #2-Series_34
mean±std - reference
mean±std - test
test
reference
Cp [ng/mL]
time [hour]
0.0
0.5
1.0
1.5
2.0
2.5
3.0
3.5
4.0
0
1
2
3
4
5
6
8
9
10
11
12
13
14
15
16
20
24
28
32
36
42
48
72

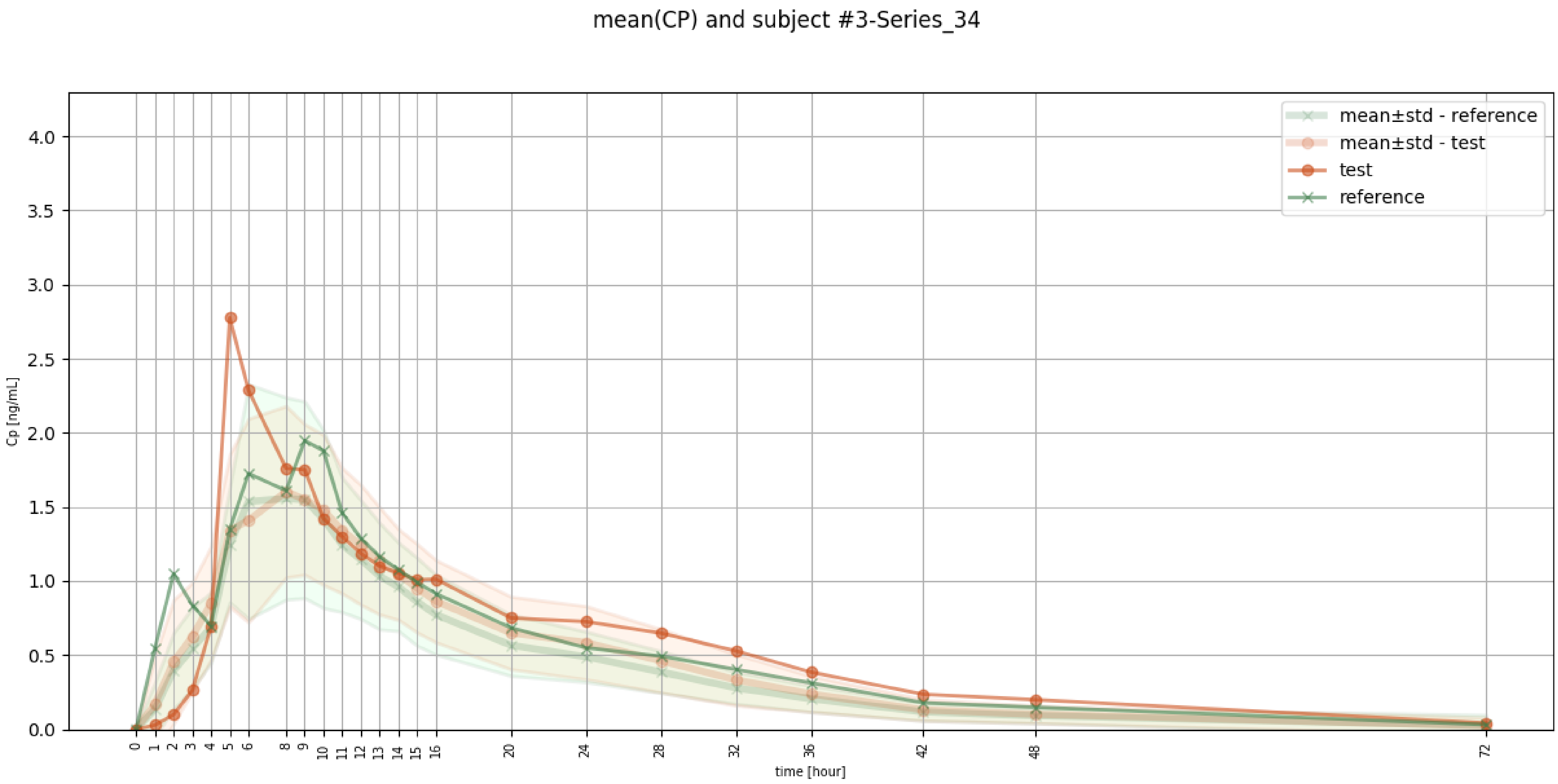

mean(CP) and subject #3-Series_34
mean±std - reference
mean±std - test
test
reference
Cp [ng/mL]
time [hour]
0.0
0.5
1.0
1.5
2.0
2.5
3.0
3.5
4.0
0 1 2 3 4 5 6 8 9 10 11 12 13 14 15 16 20 24 28 32 36 42 48 72

mean(CP) and subject #5-Series_34

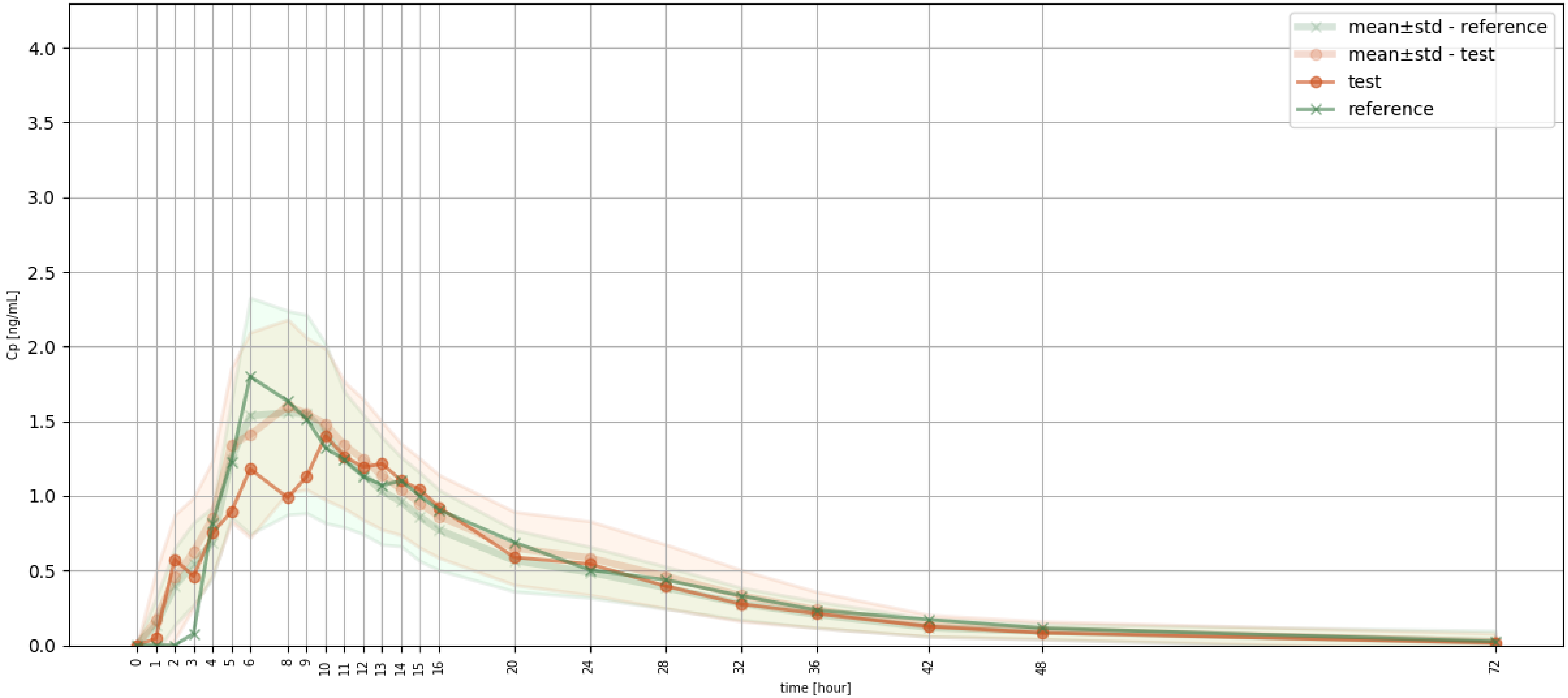

mean(CP) and subject #6-Series_34

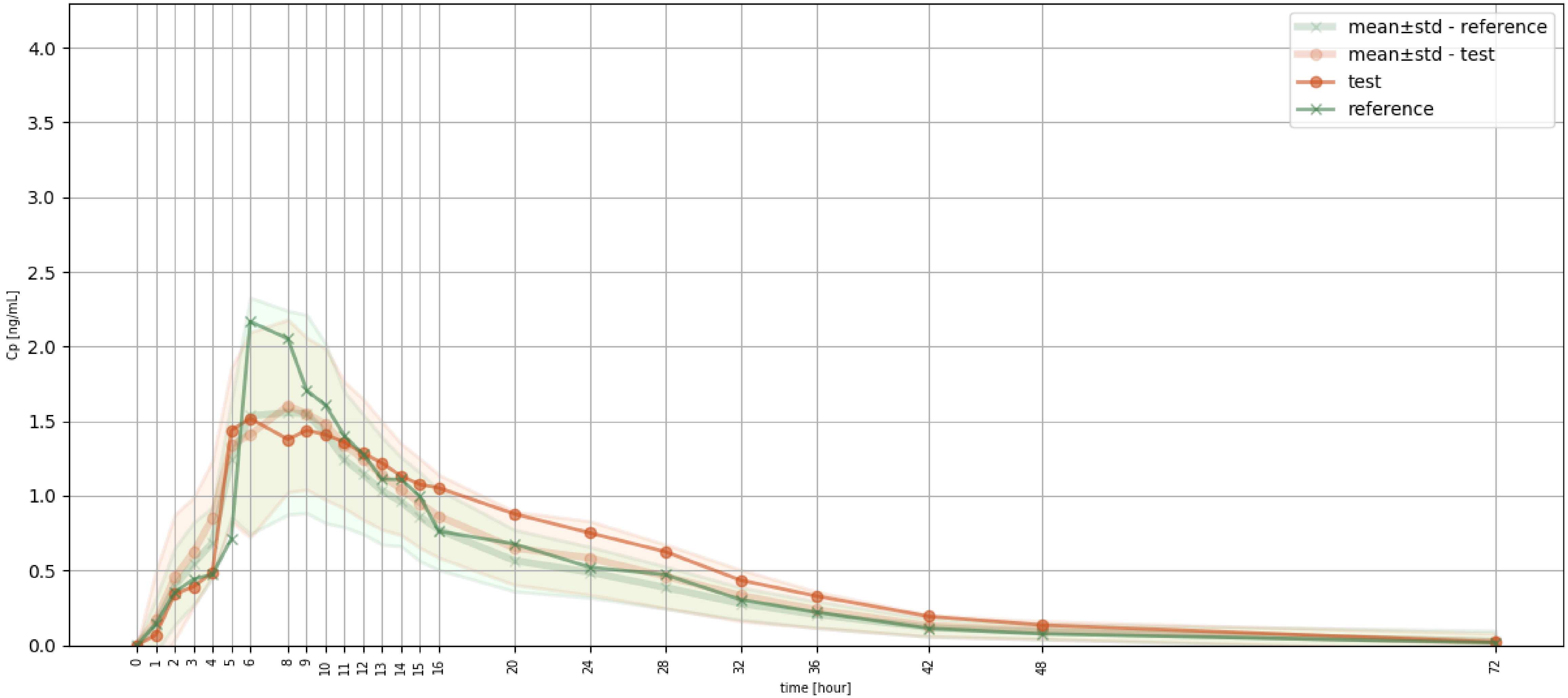

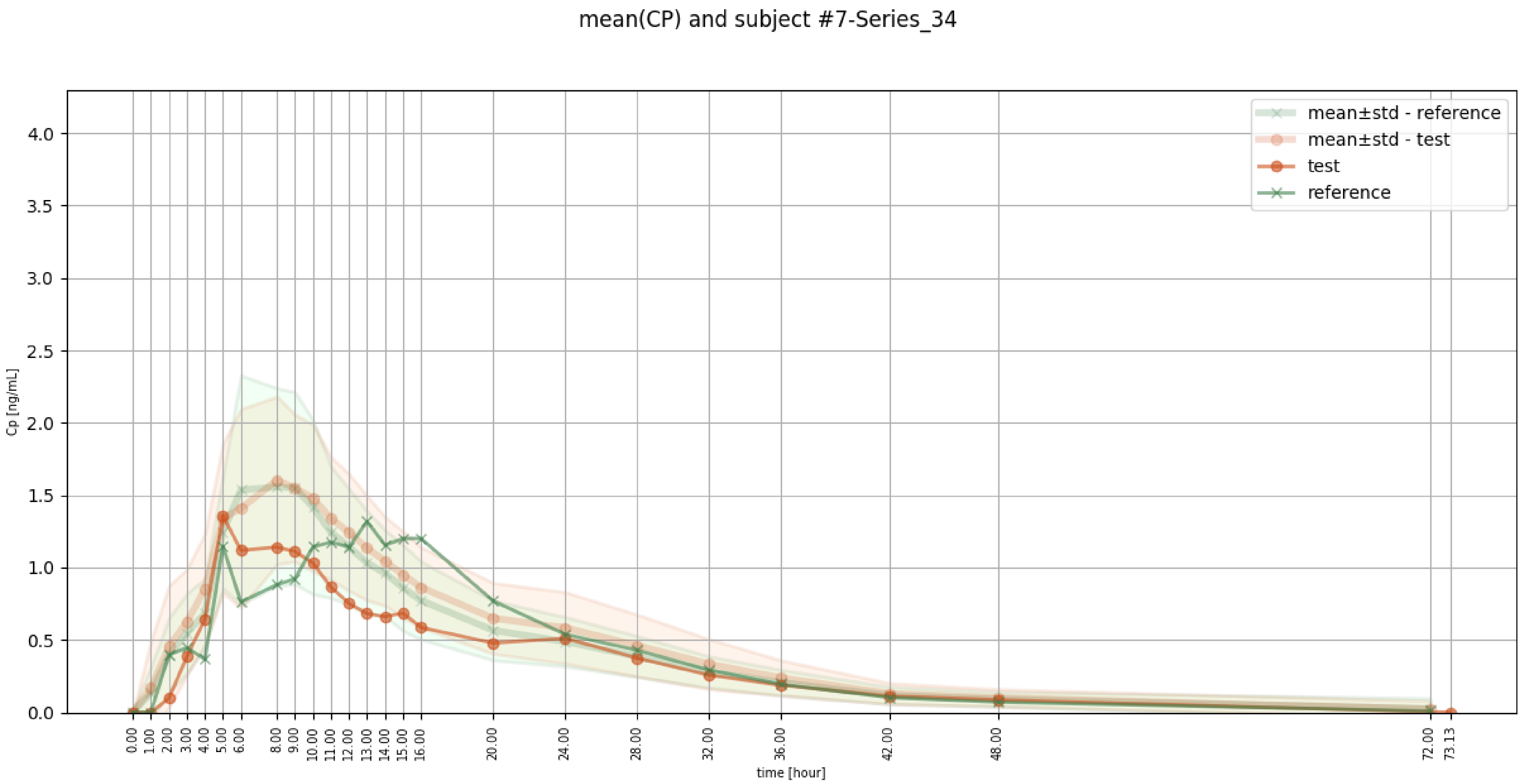
mean(CP) and subject #7-Series_34
mean±std - reference
mean±std - test
test
reference
Cp [ng/mL]
time [hour]
0.0
0.5
1.0
1.5
2.0
2.5
3.0
3.5
4.0
0.00
1.00
2.00
3.00
4.00
5.00
6.00
8.00
9.00
10.00
11.00
12.00
13.00
14.00
15.00
16.00
20.00
24.00
28.00
32.00
36.00
42.00
48.00
72.00
73.13

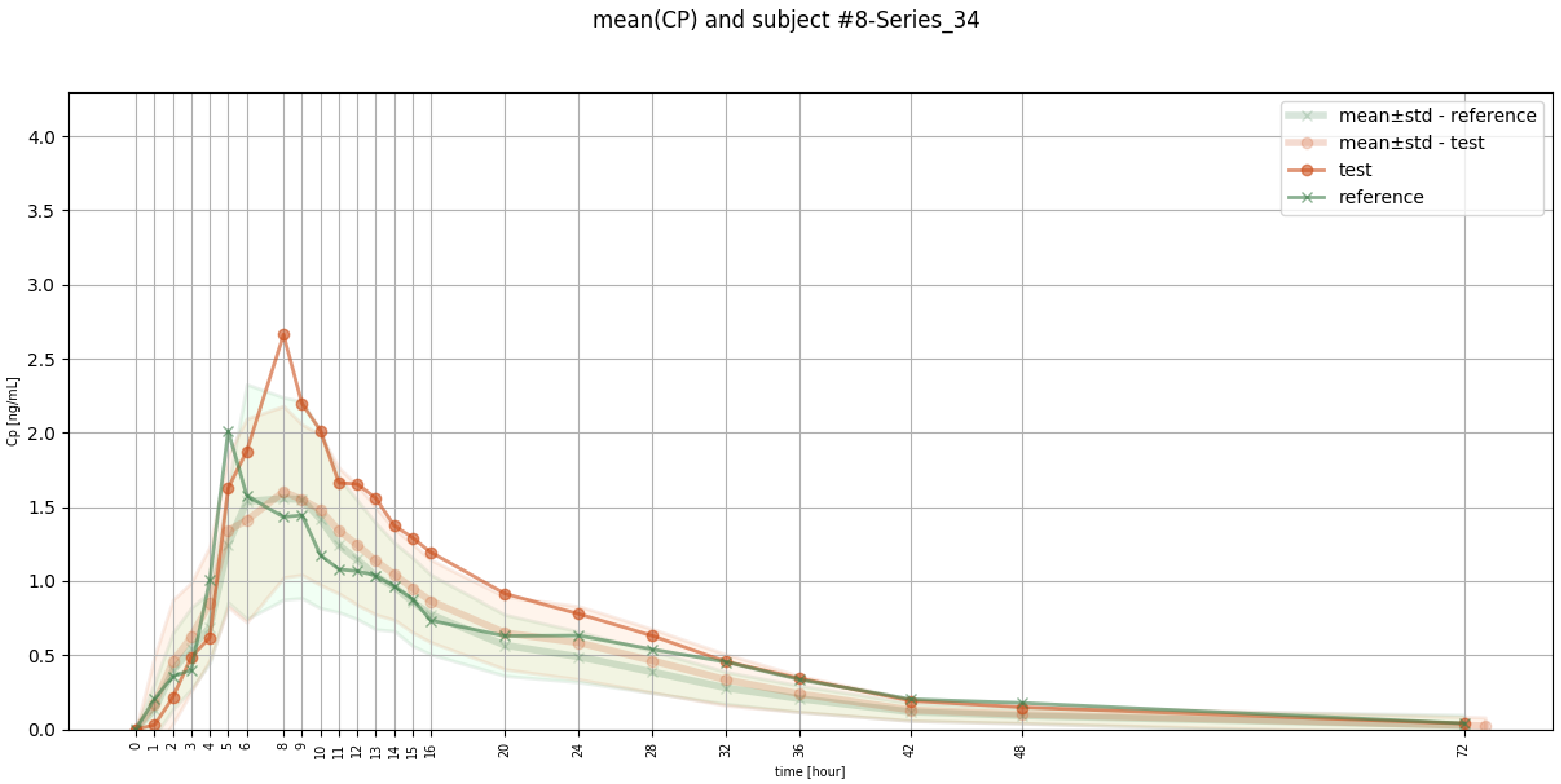

mean(CP) and subject #8-Series_34
mean±std - reference
mean±std - test
test
reference
Cp [ng/mL]
time [hour]
4.0
3.5
3.0
2.5
2.0
1.5
1.0
0.5
0.0
0 1 2 3 4 5 6 8 9 10 11 12 13 14 15 16 20 24 28 32 36 42 48 72

mean(CP) and subject #11-Series_34

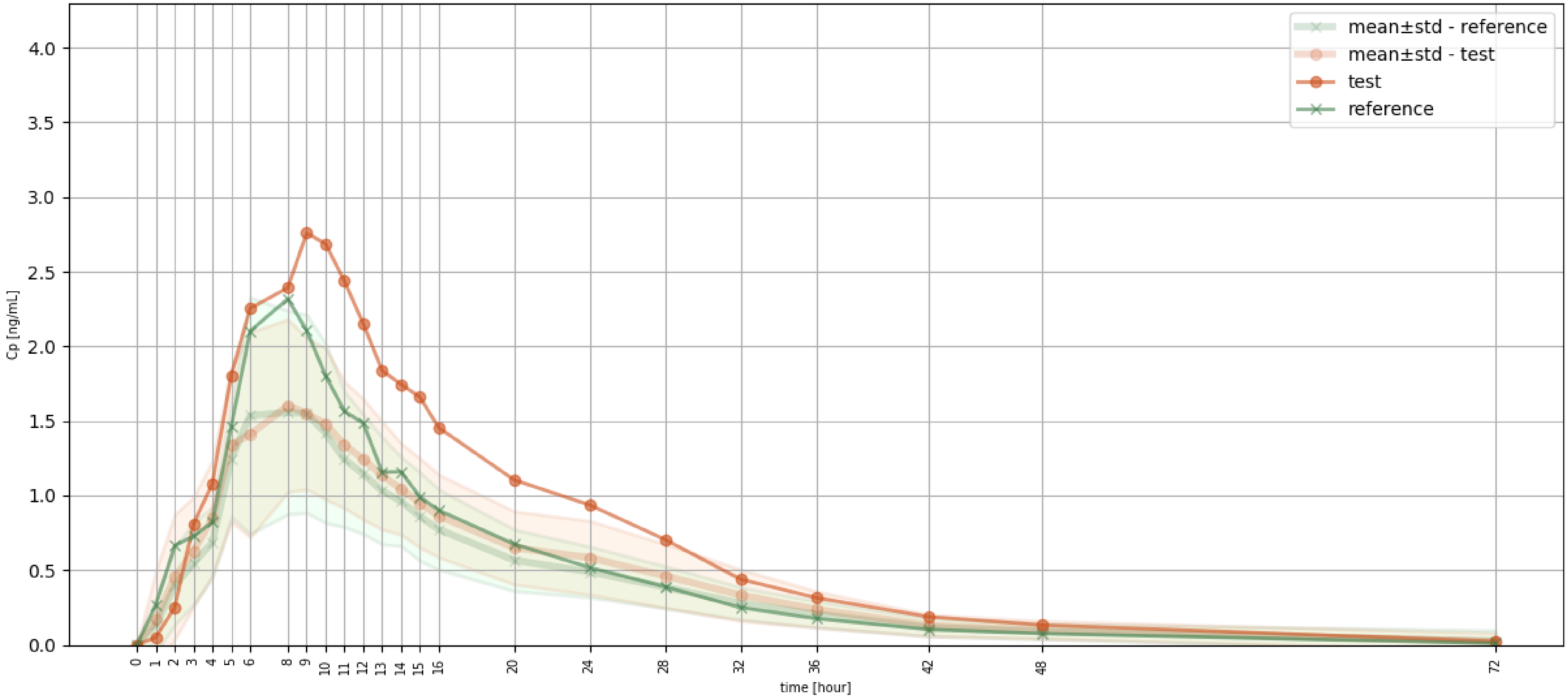

mean(CP) and subject #12-Series_34

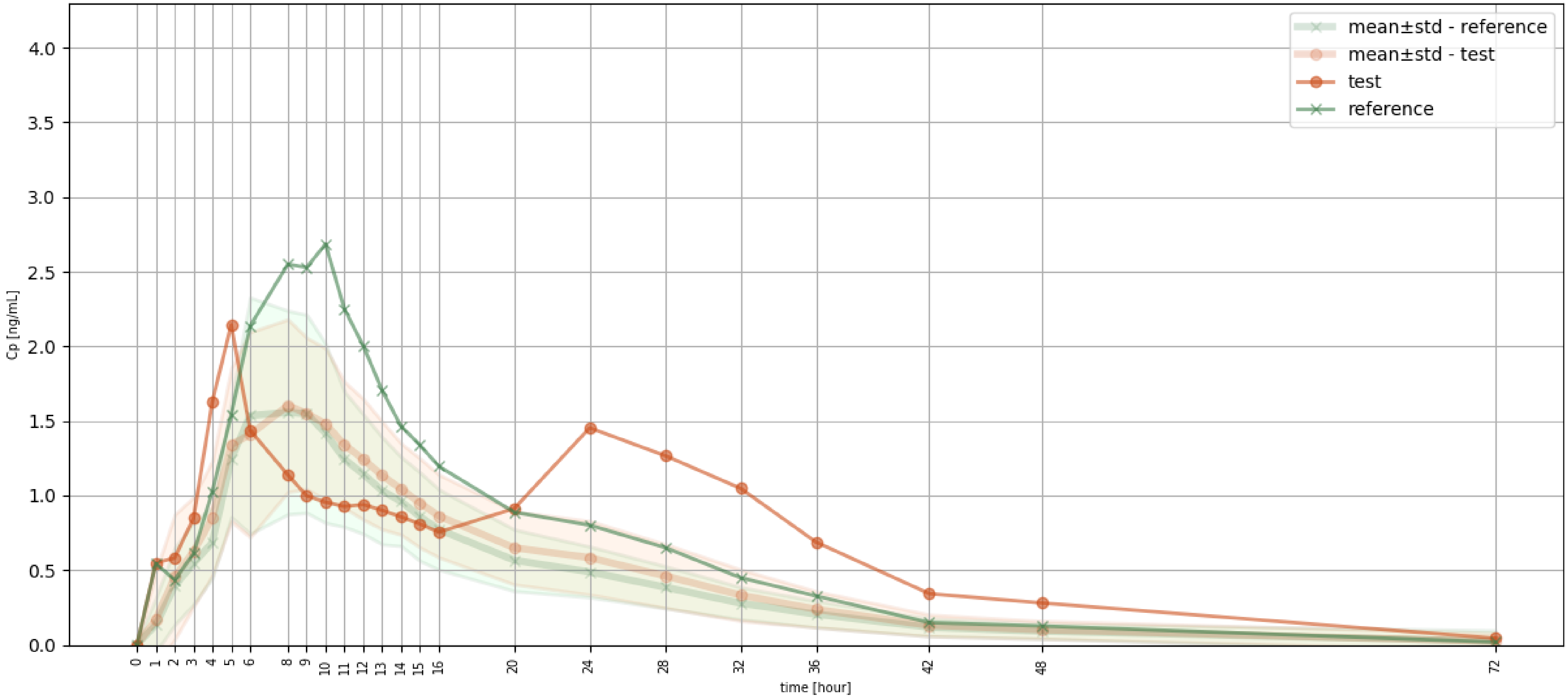

mean(CP) and subject #13-Series_34

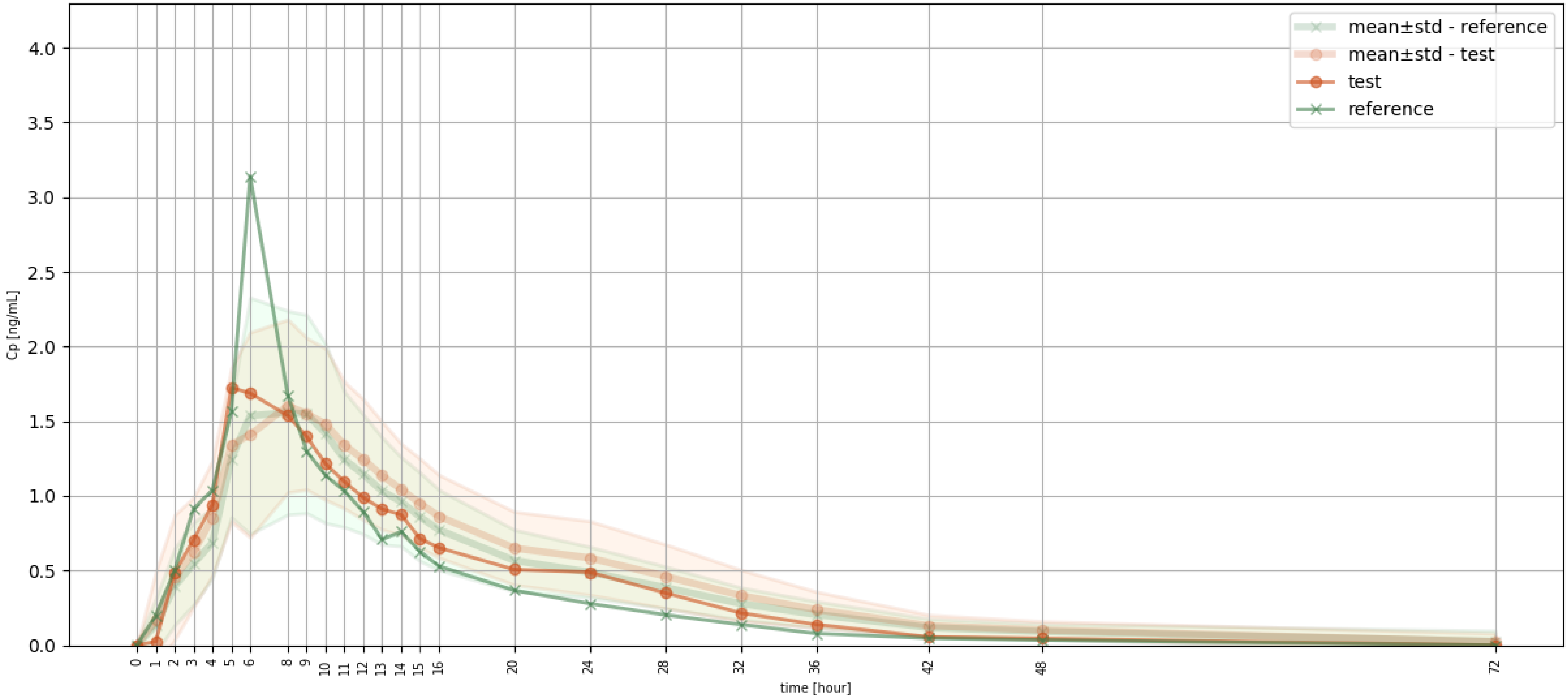

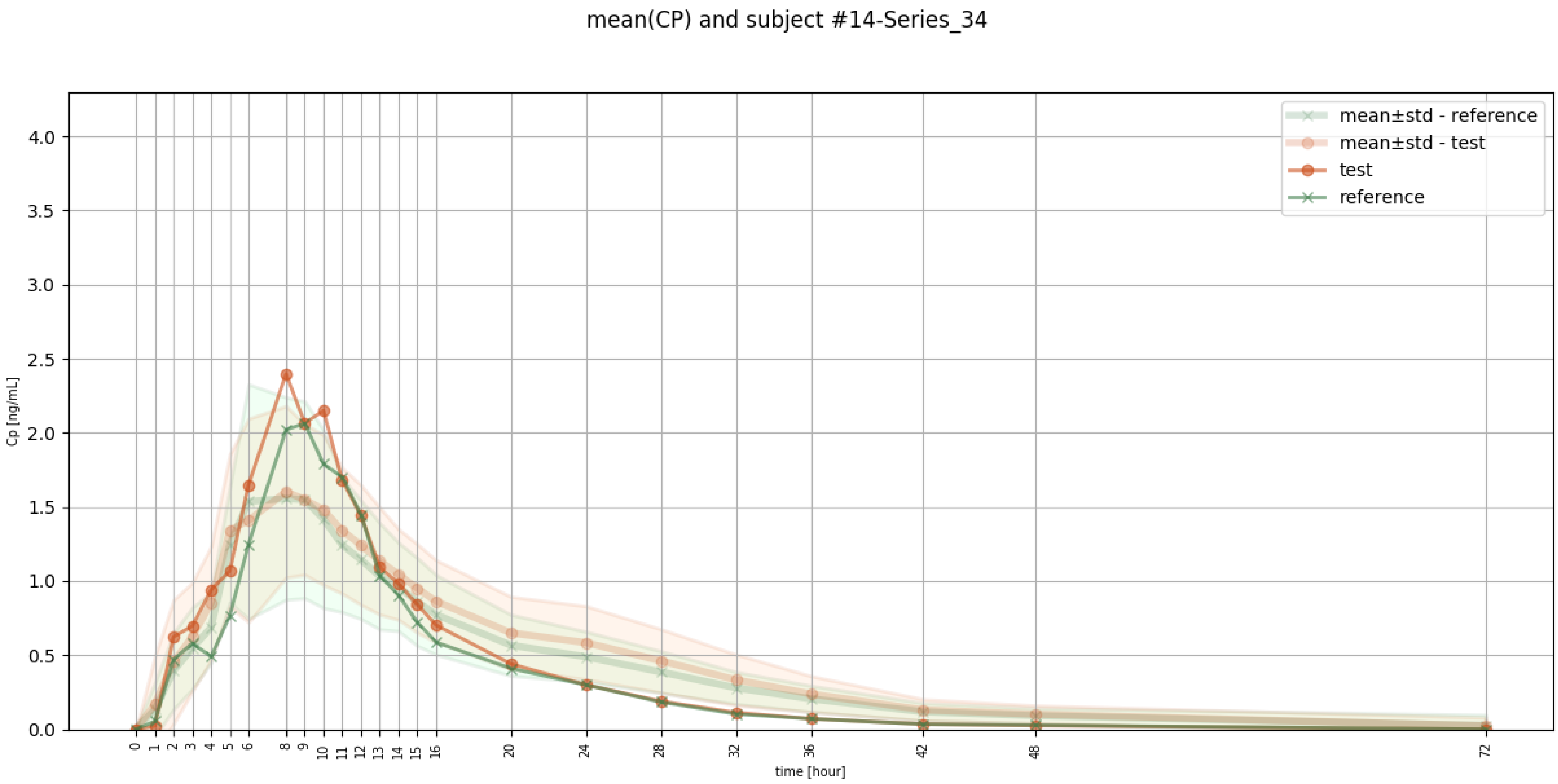

mean(CP) and subject #14-Series_34
mean±std - reference
mean±std - test
test
reference
Cp [ng/mL]
time [hour]
0.0
0.5
1.0
1.5
2.0
2.5
3.0
3.5
4.0
0 1 2 3 4 5 6 8 9 10 11 12 13 14 15 16 20 24 28 32 36 42 48 72

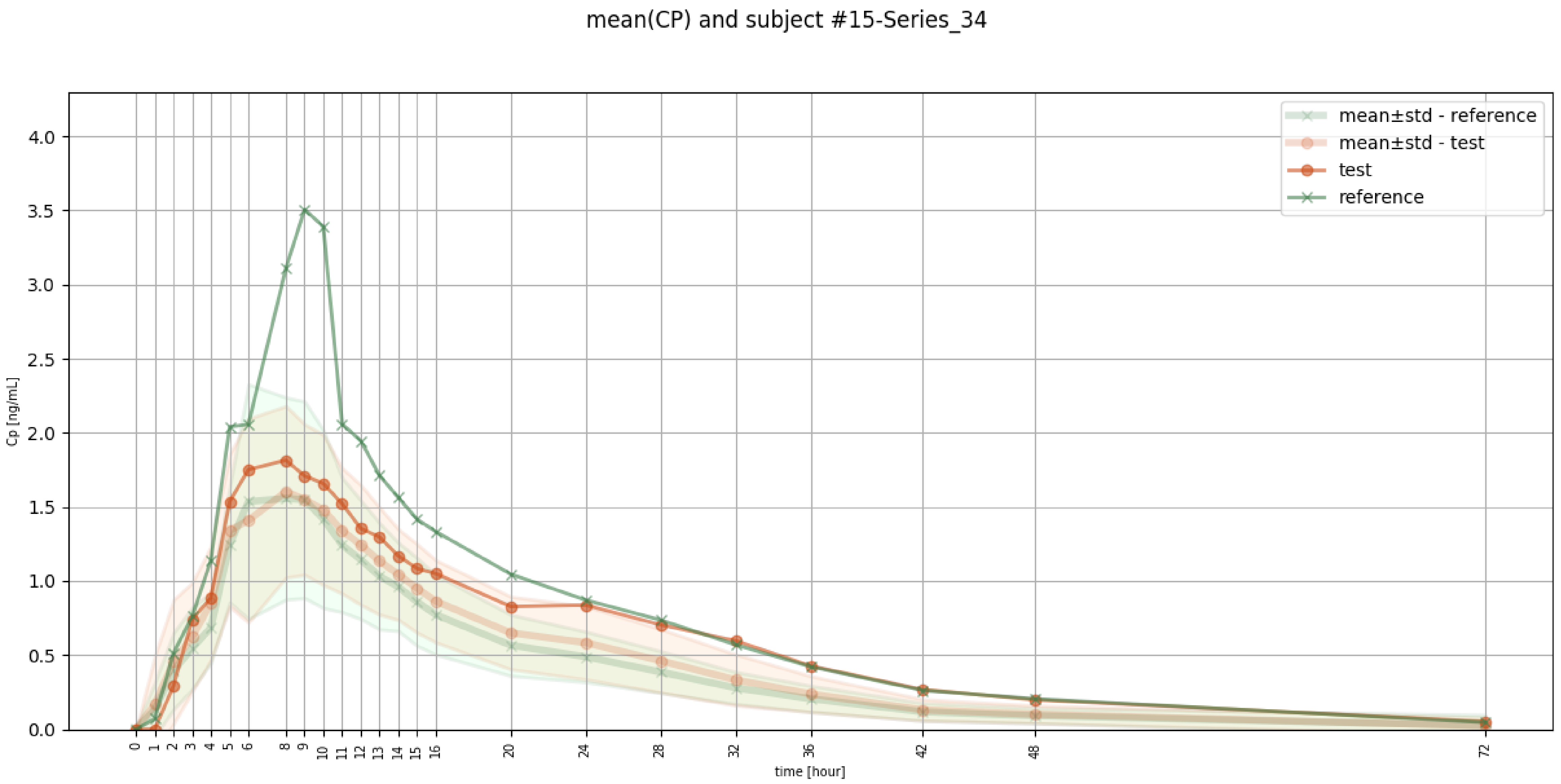
mean(CP) and subject #15-Series_34
mean±std - reference
mean±std - test
test
reference
Cp [ng/mL]
time [hour]
0.0
0.5
1.0
1.5
2.0
2.5
3.0
3.5
4.0
0
1
2
3
4
5
6
8
9
10
11
12
13
14
15
16
20
24
28
32
36
42
48
72

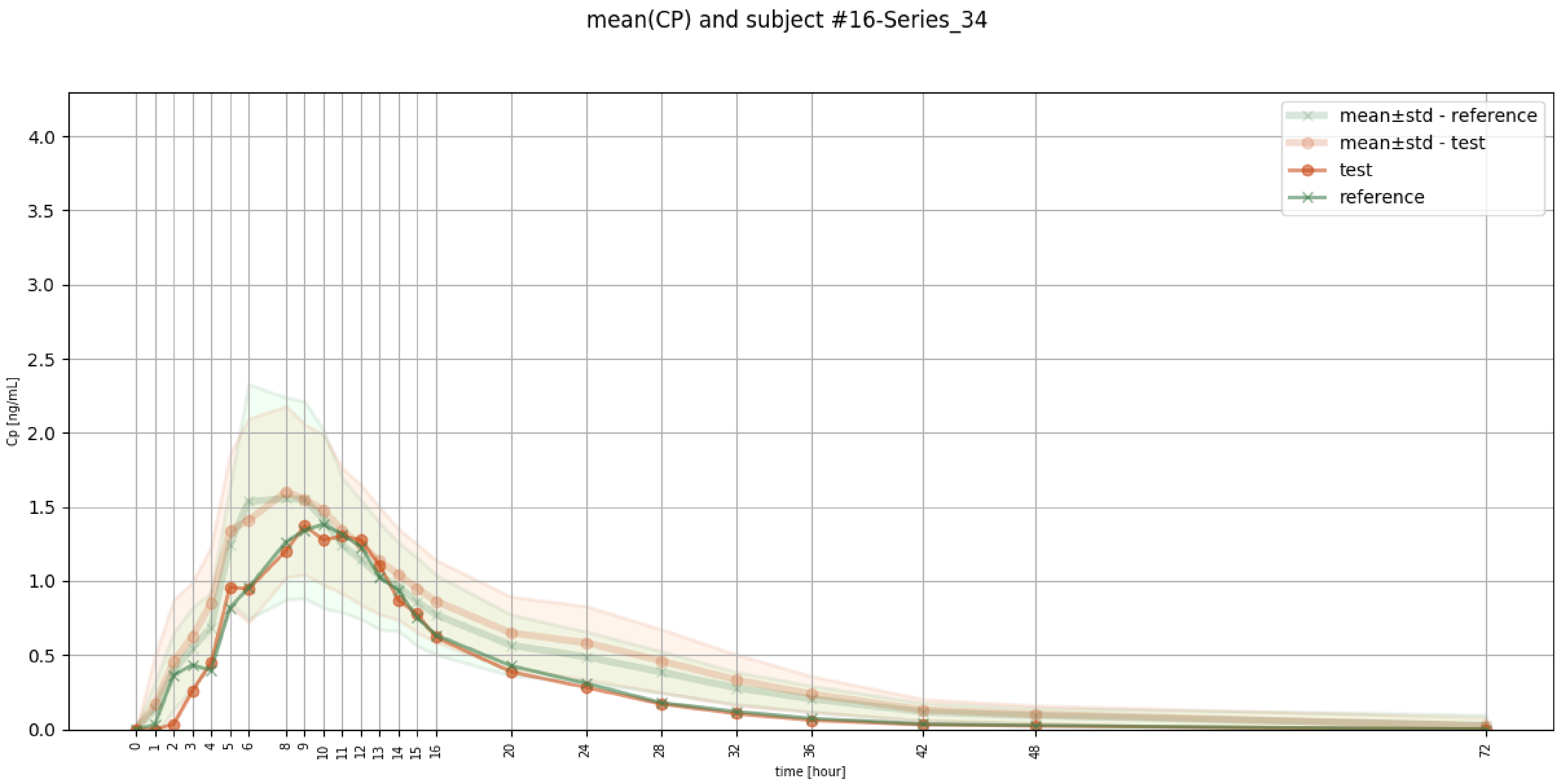

mean(CP) and subject #16-Series_34
mean±std - reference
mean±std - test
test
reference
Cp [ng/mL]
time [hour]
0.0
0.5
1.0
1.5
2.0
2.5
3.0
3.5
4.0
0
1
2
3
4
5
6
8
9
10
11
12
13
14
15
16
20
24
28
32
36
42
48
72

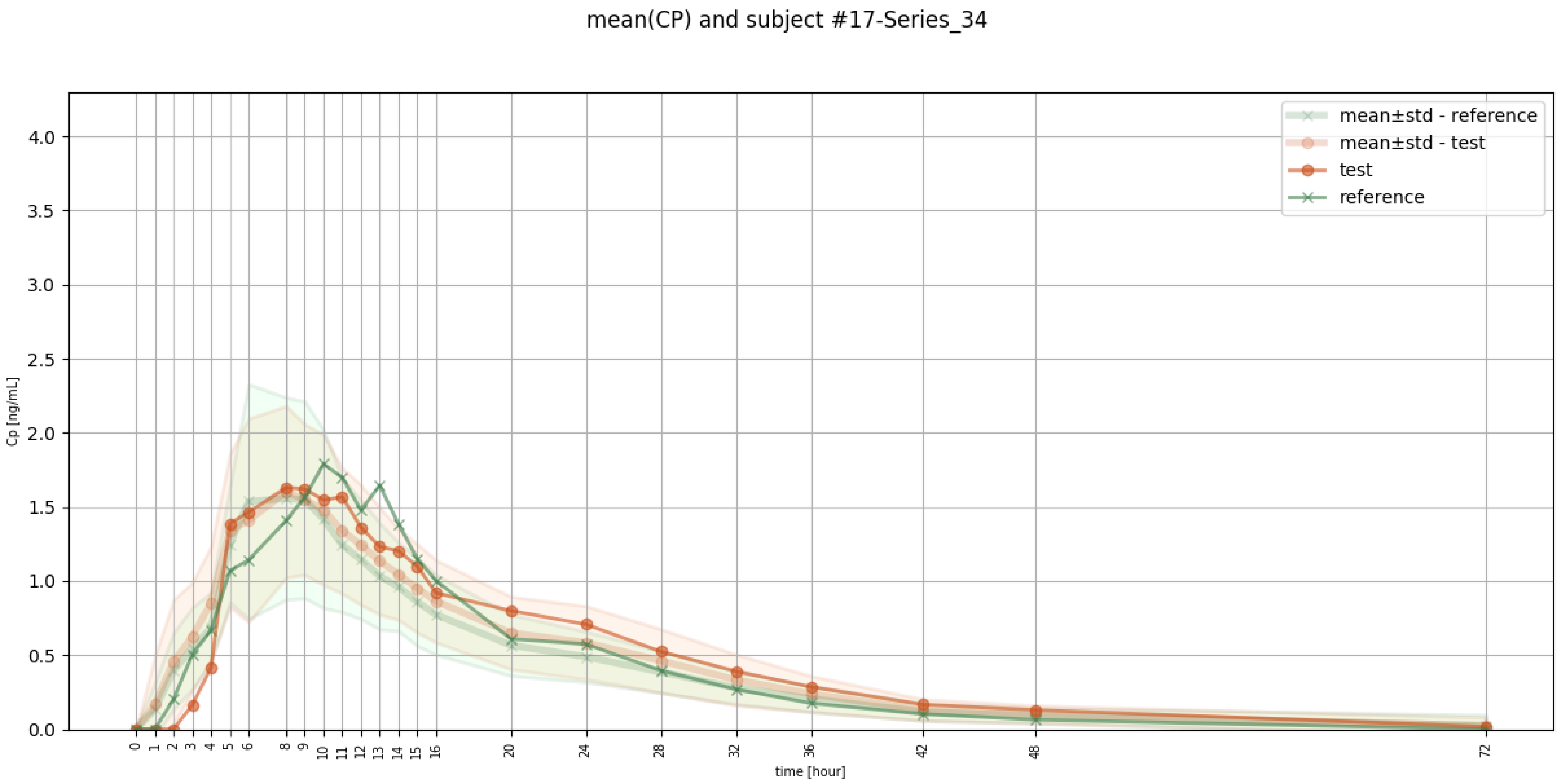

mean(CP) and subject #17-Series_34
mean±std - reference
mean±std - test
test
reference
Cp [ng/mL]
time [hour]
0.0
0.5
1.0
1.5
2.0
2.5
3.0
3.5
4.0
0 1 2 3 4 5 6 8 9 10 11 12 13 14 15 16 20 24 28 32 36 42 48 72

mean(CP) and subject #19-Series_34

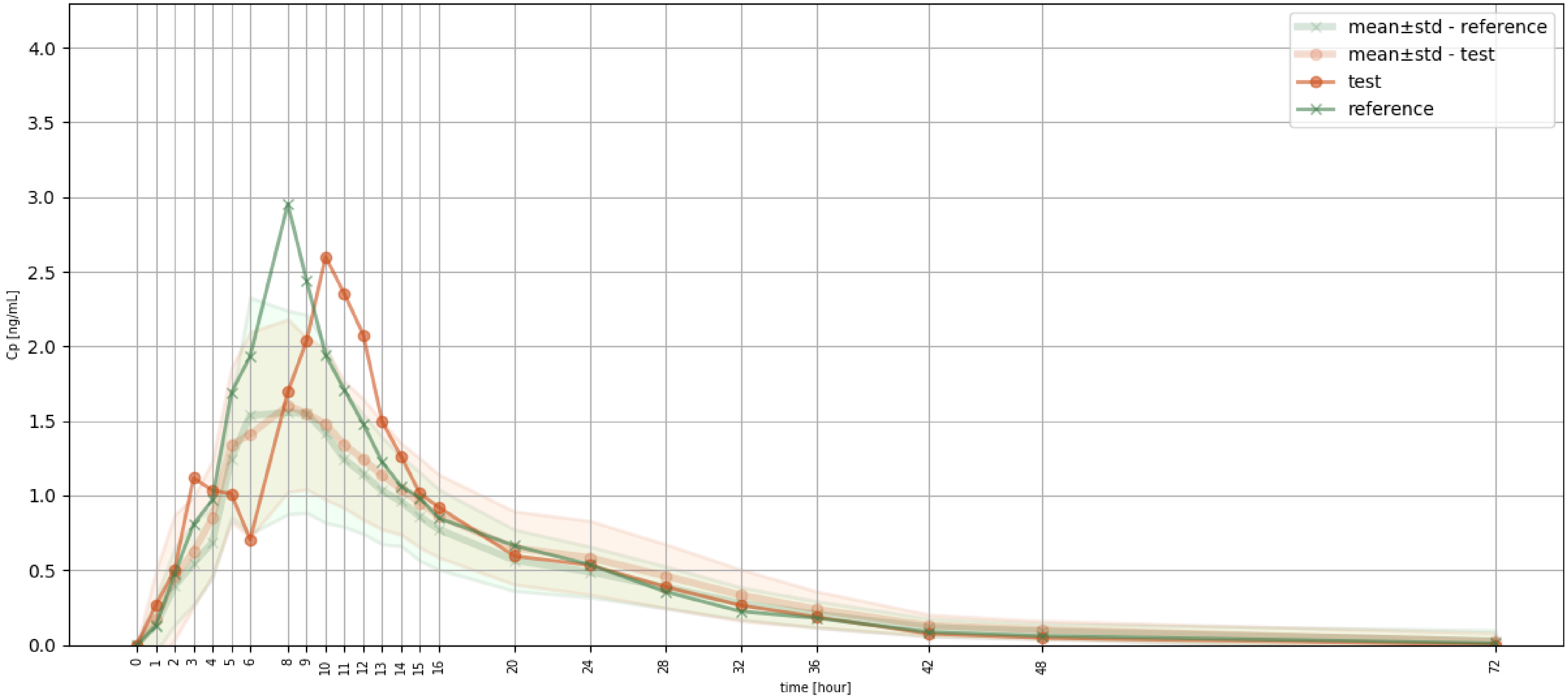

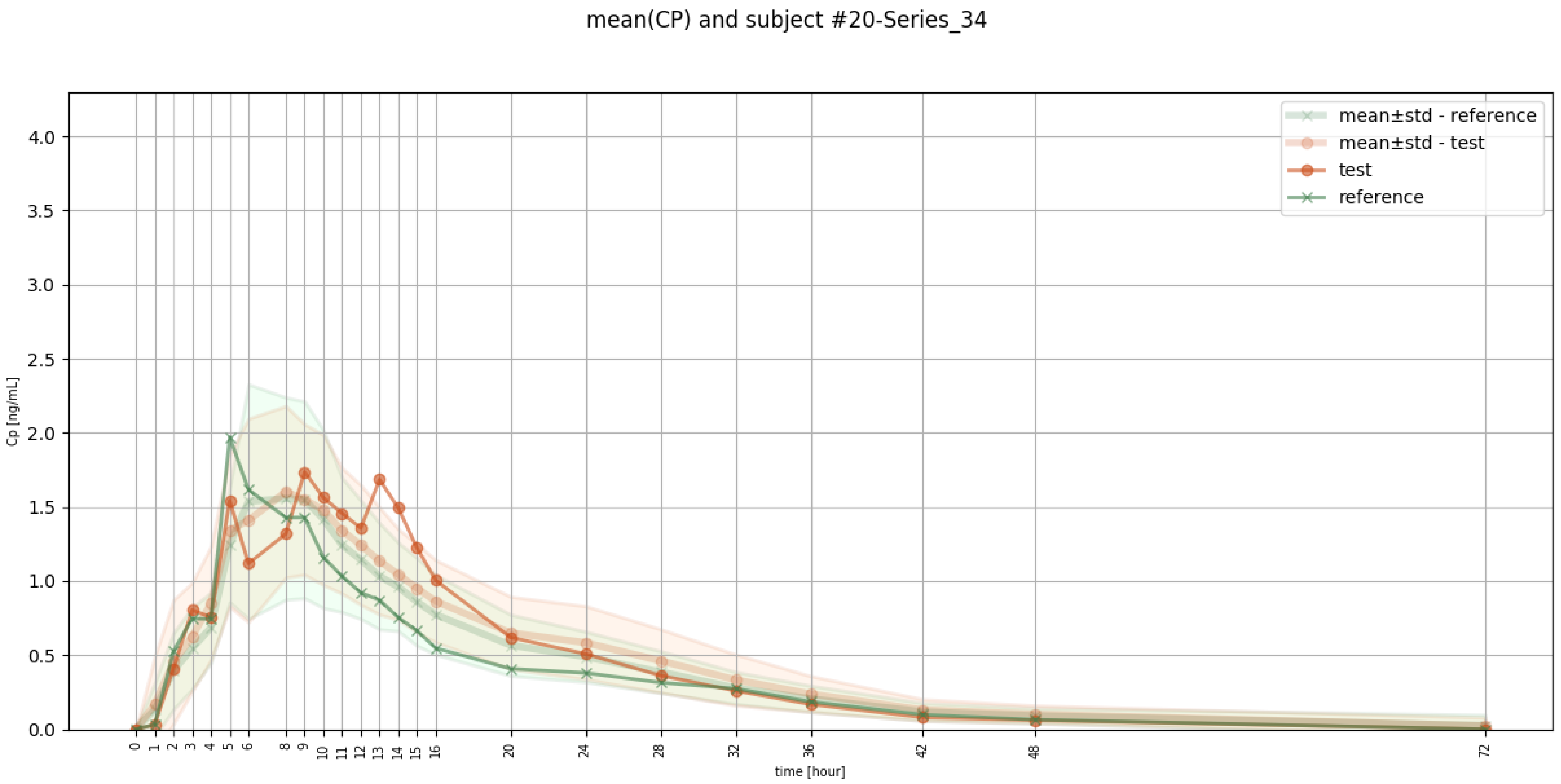

mean(CP) and subject #20-Series_34
mean±std - reference
mean±std - test
test
reference
Cp [ng/mL]
time [hour]
0.0
0.5
1.0
1.5
2.0
2.5
3.0
3.5
4.0
0
1
2
3
4
5
6
8
9
10
11
12
13
14
15
16
20
24
28
32
36
42
48
72

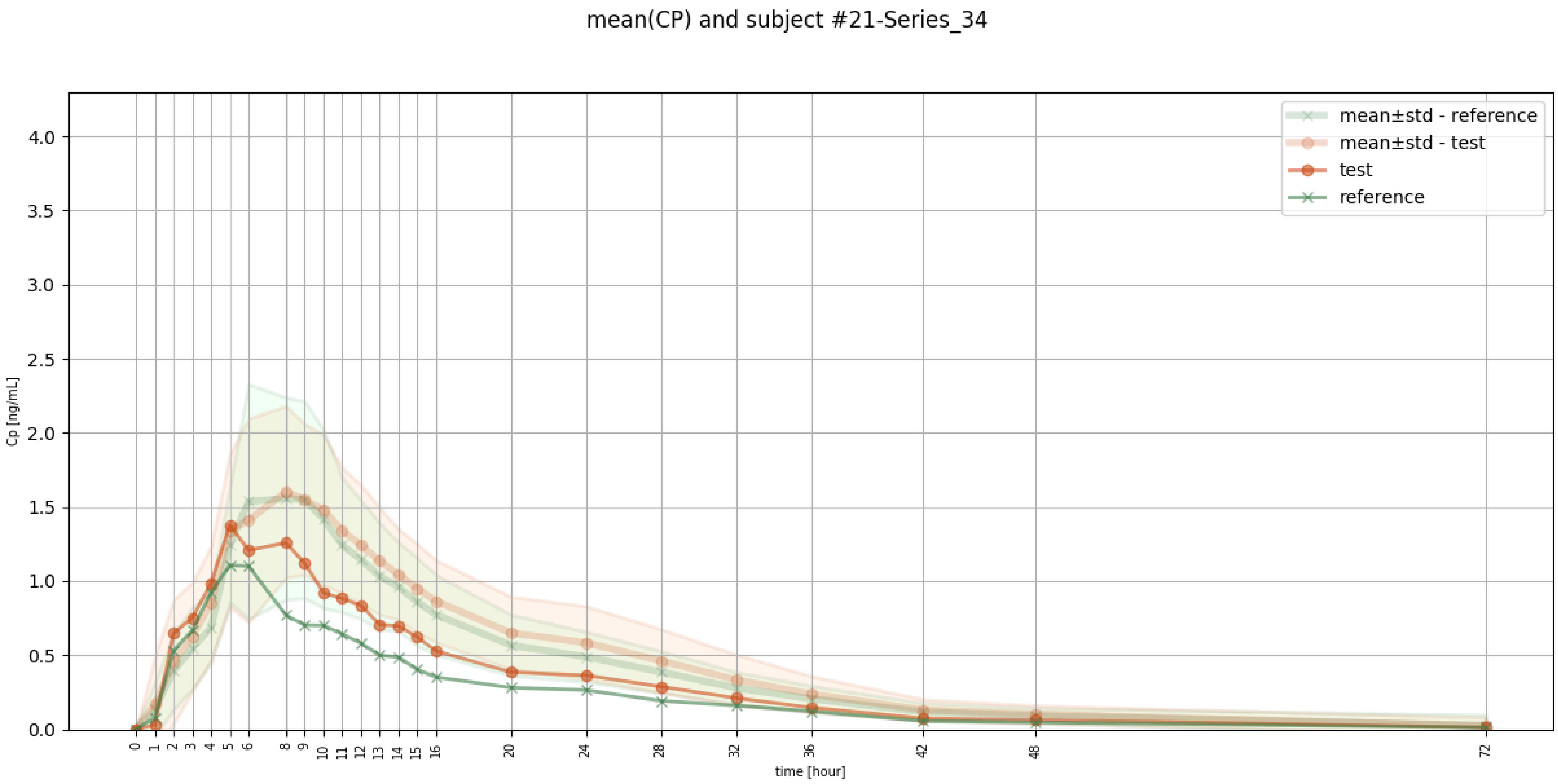

mean(CP) and subject #21-Series_34
mean±std - reference
mean±std - test
test
reference
Cp [ng/mL]
4.0
3.5
3.0
2.5
2.0
1.5
1.0
0.5
0.0
0
1
2
3
4
5
6
8
9
10
11
12
13
14
15
16
20
24
28
32
36
42
48
72
time [hour]

mean(CP) and subject #22-Series_34

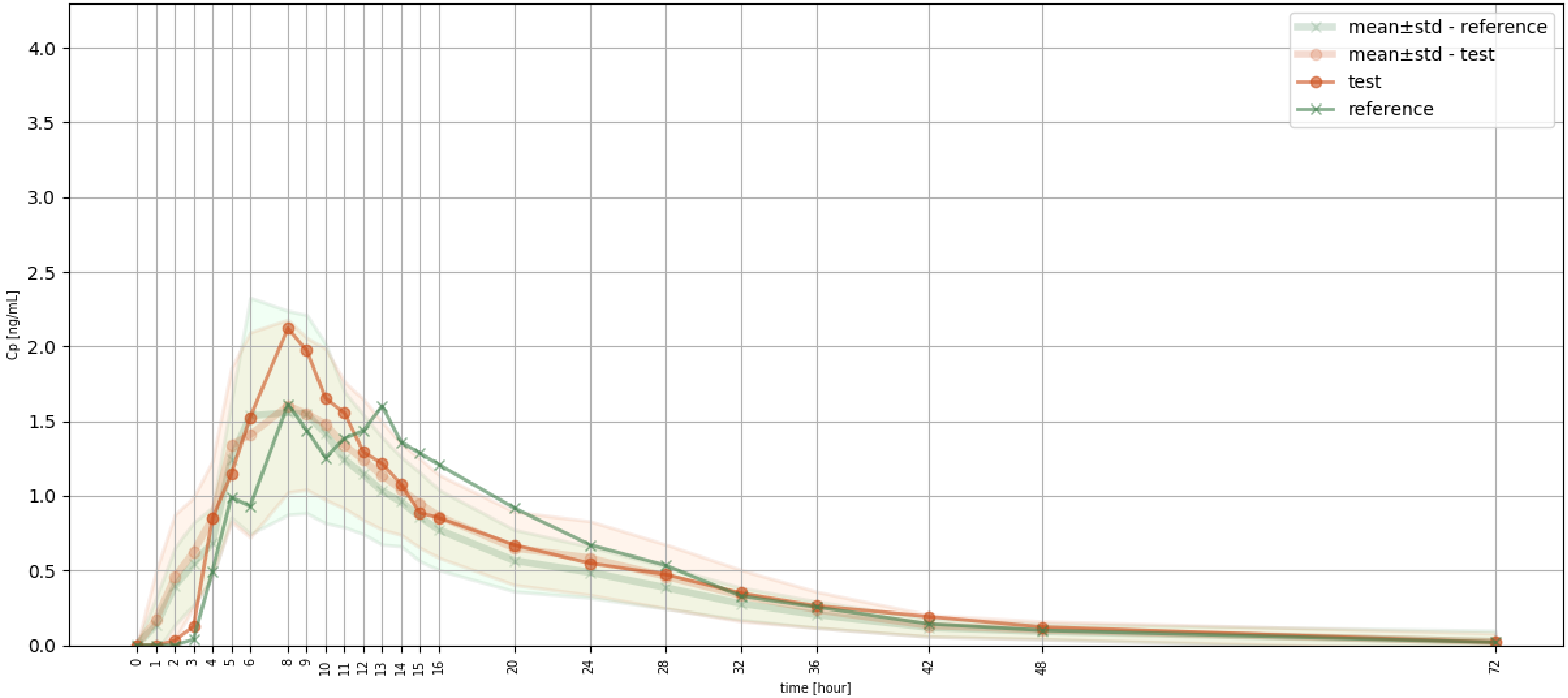

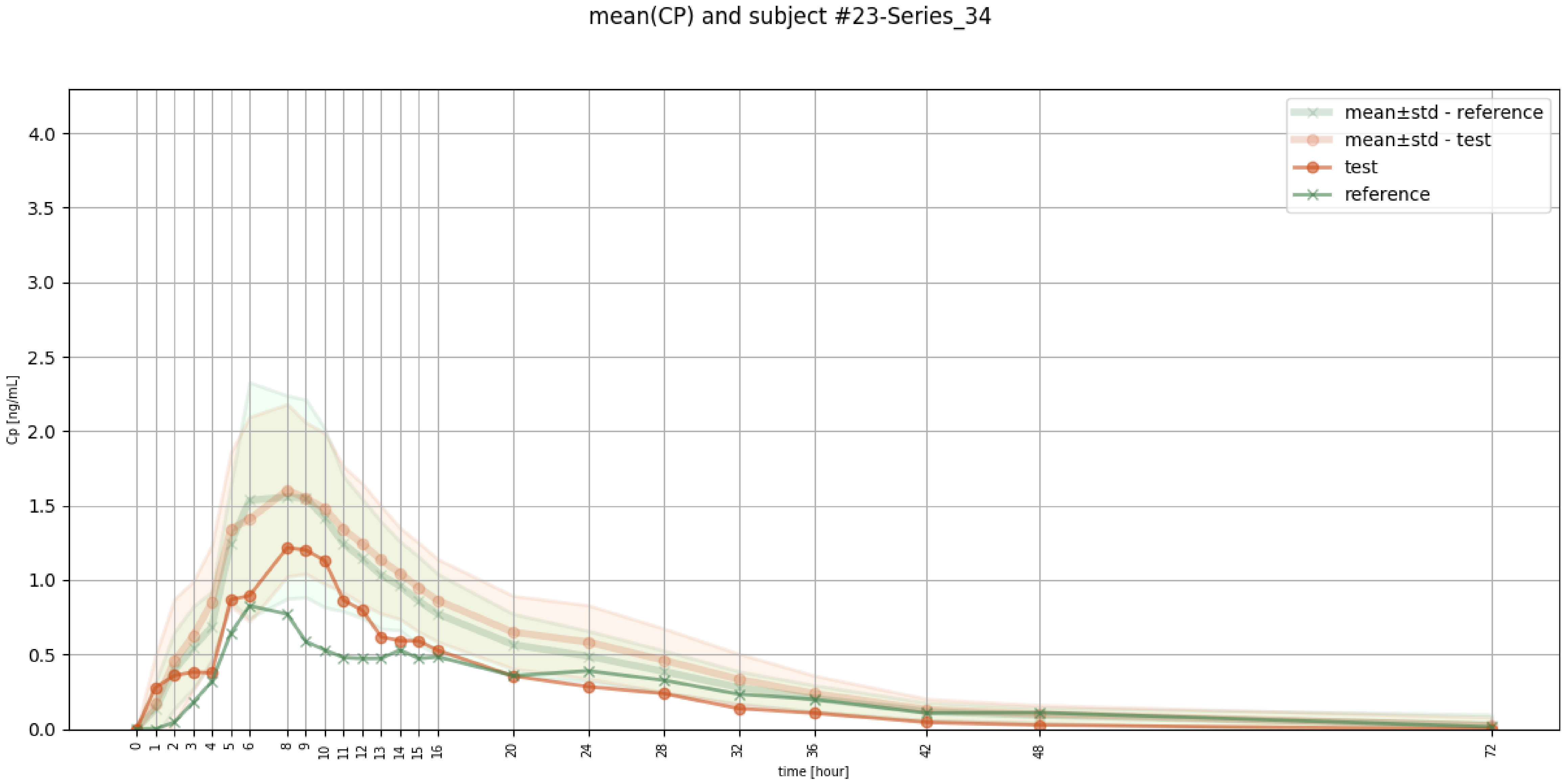

mean(CP) and subject #23-Series_34
mean±std - reference
mean±std - test
test
reference
Cp [ng/mL]
4.0
3.5
3.0
2.5
2.0
1.5
1.0
0.5
0.0
0
1
2
3
4
5
6
8
9
10
11
12
13
14
15
16
20
24
28
32
36
42
48
72
time [hour]

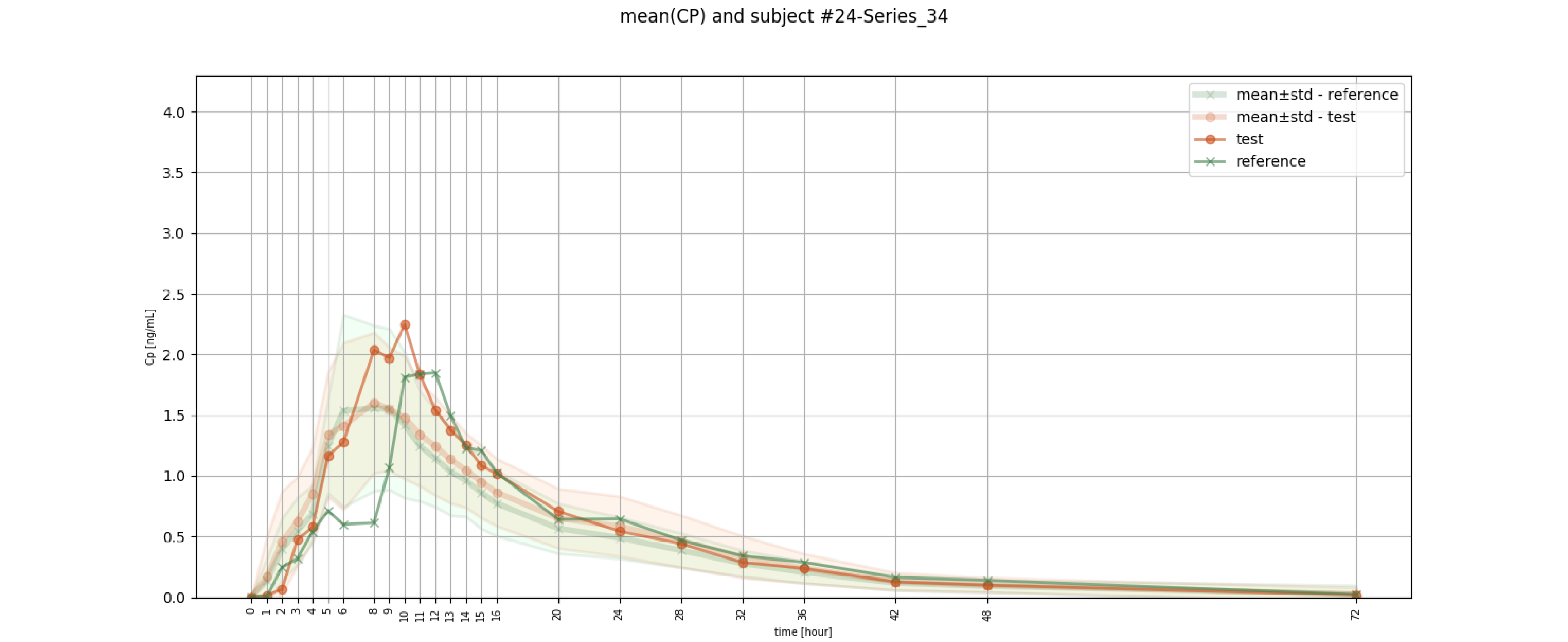
mean(CP) and subject #24-Series_34
mean±std - reference
mean±std - test
test
reference
Cp [ng/mL]
0.0
0.5
1.0
1.5
2.0
2.5
3.0
3.5
4.0
time [hour]
0
1
2
3
4
5
6
8
9
10
11
12
13
14
15
16
20
24
28
32
36
42
48
72

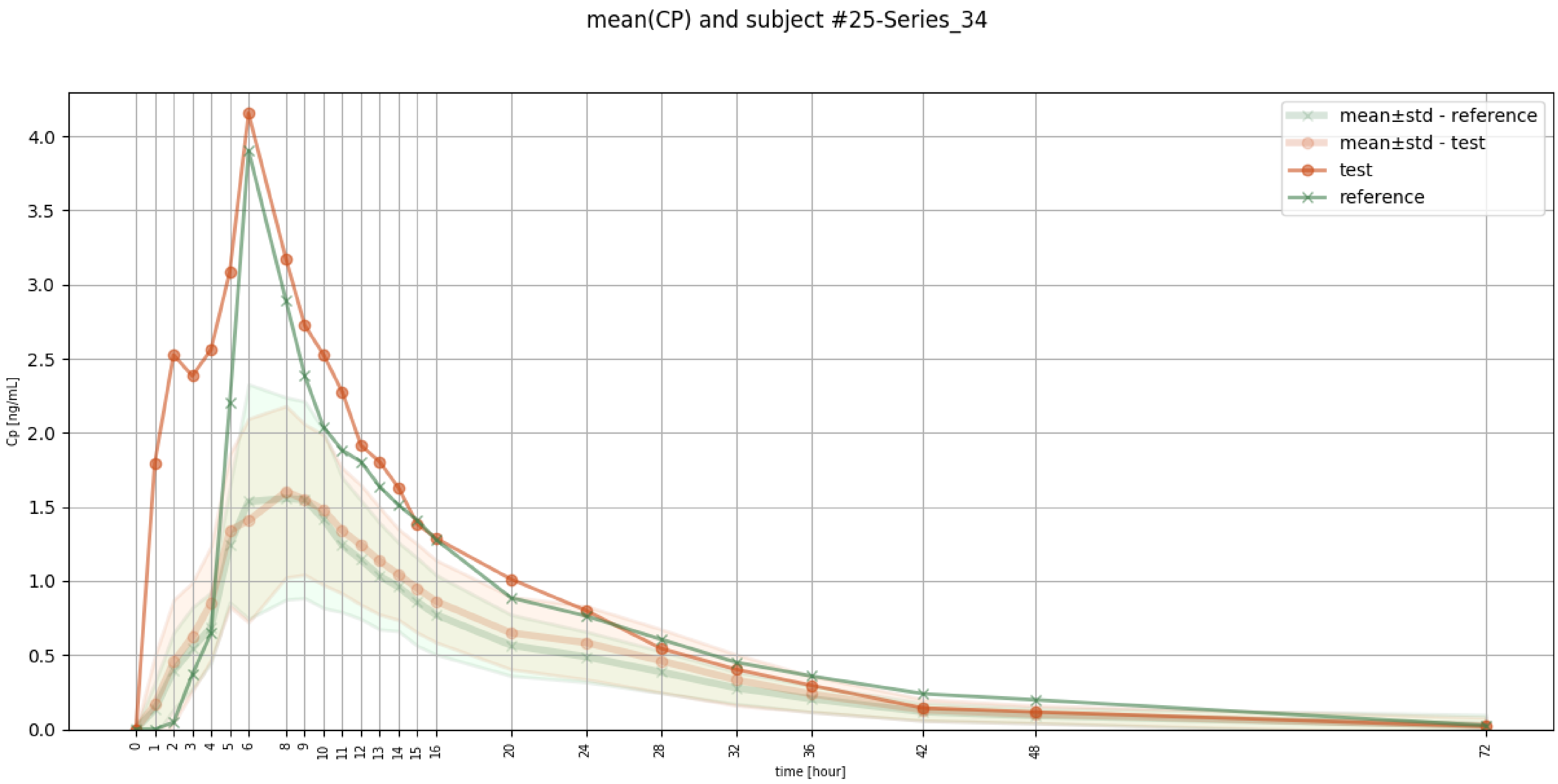

mean(CP) and subject #25-Series_34
mean±std - reference
mean±std - test
test
reference
Cp [ng/mL]
time [hour]
0.0
0.5
1.0
1.5
2.0
2.5
3.0
3.5
4.0
0
1
2
3
4
5
6
8
9
10
11
12
13
14
15
16
20
24
28
32
36
42
48
72

mean(CP) and subject #26-Series_34

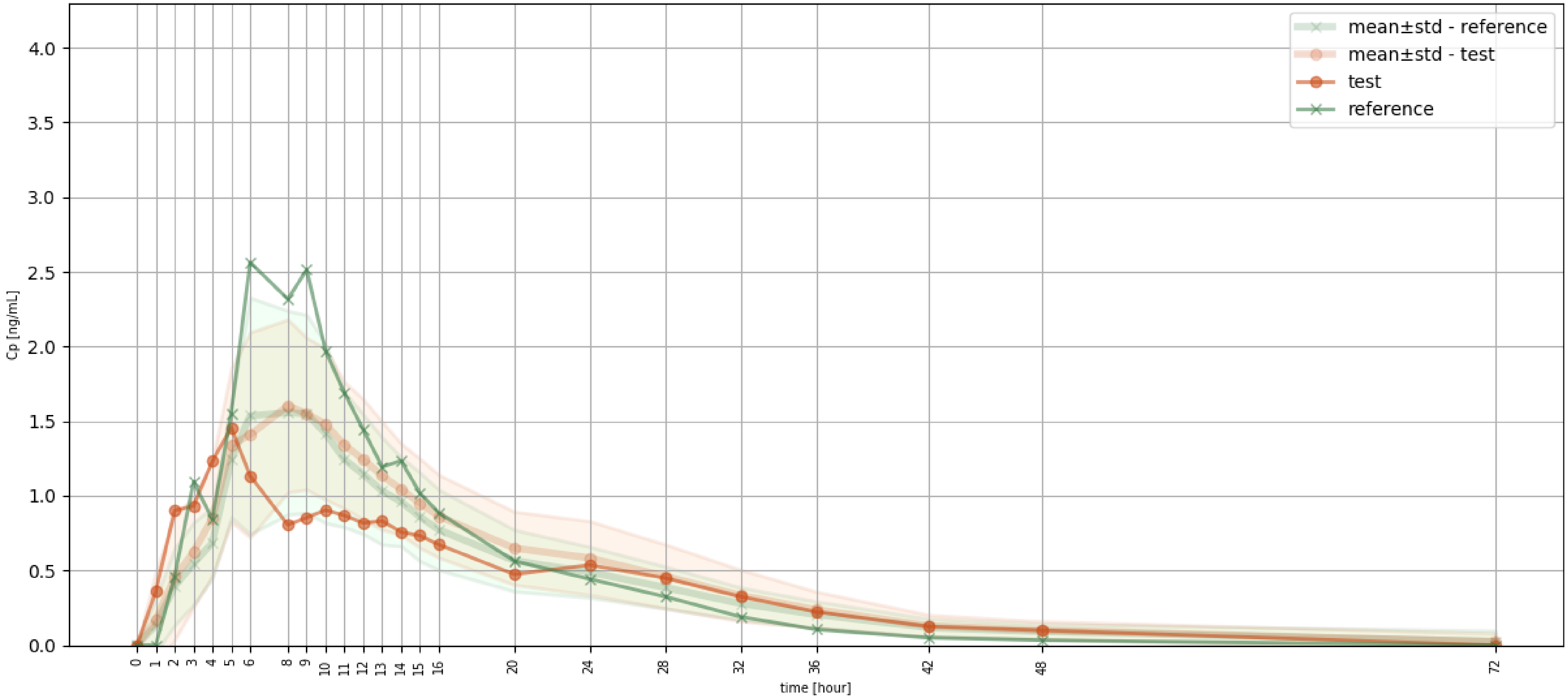

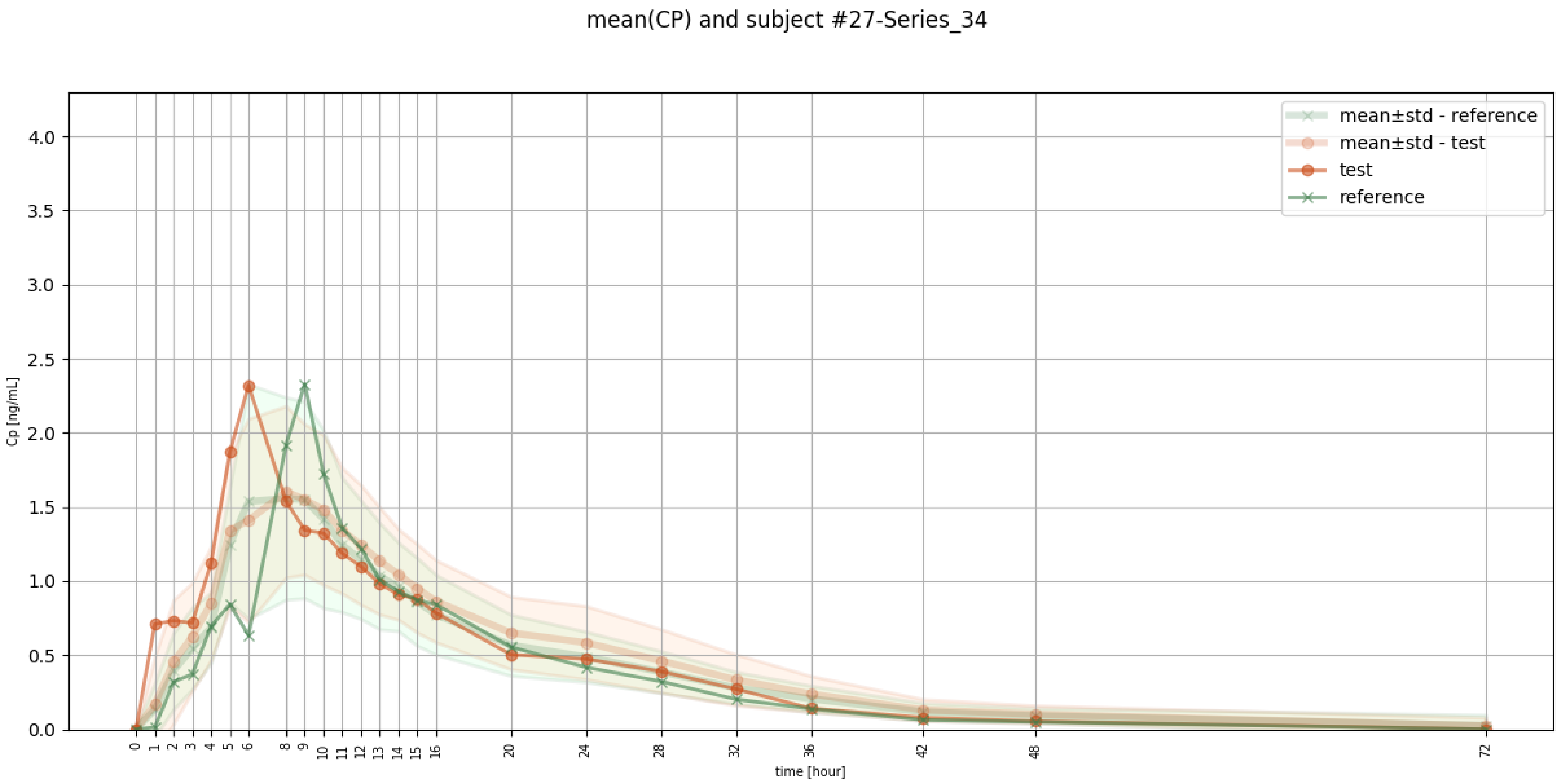

mean(CP) and subject #27-Series_34
mean±std - reference
mean±std - test
test
reference
Cp [ng/mL]
time [hour]
0.0
0.5
1.0
1.5
2.0
2.5
3.0
3.5
4.0
0
1
2
3
4
5
6
8
9
10
11
12
13
14
15
16
20
24
28
32
36
42
48
72

mean(CP) and subject #28-Series_34

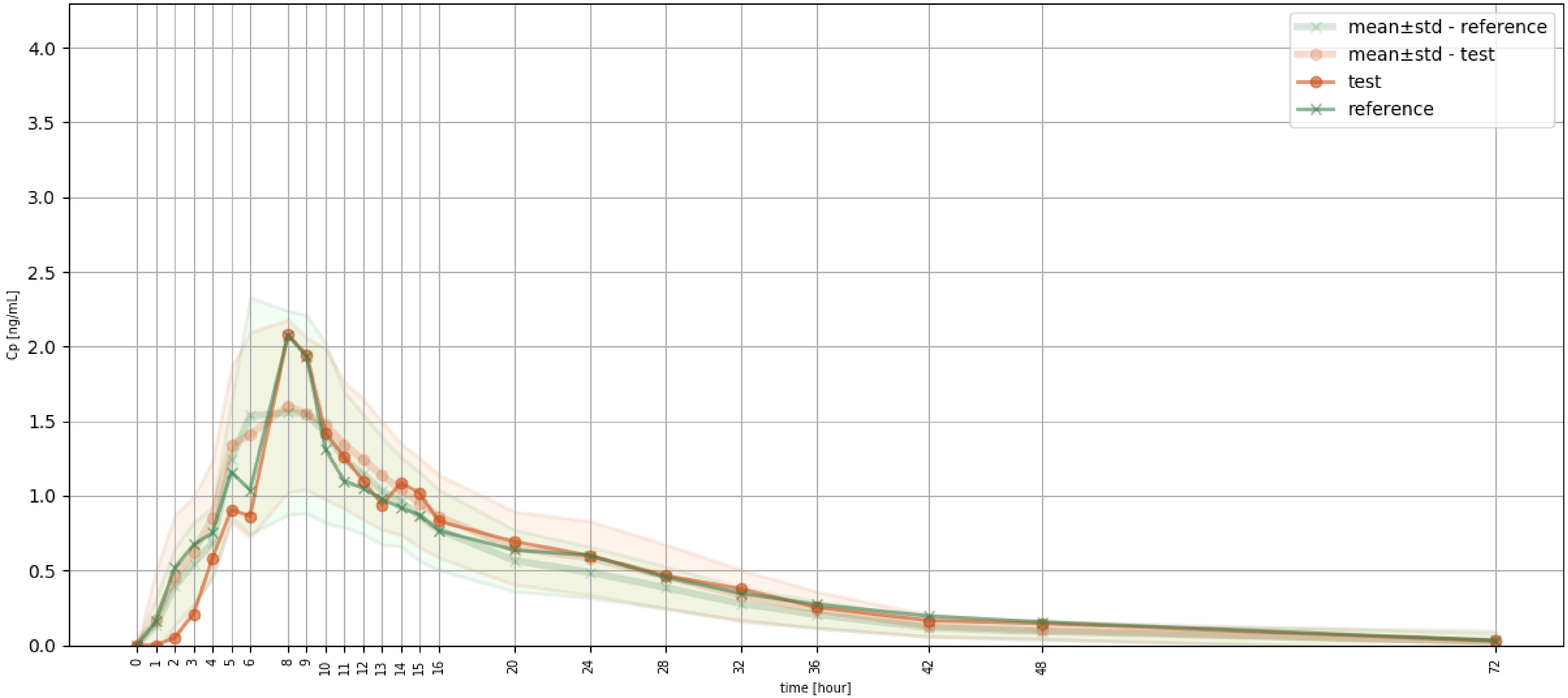

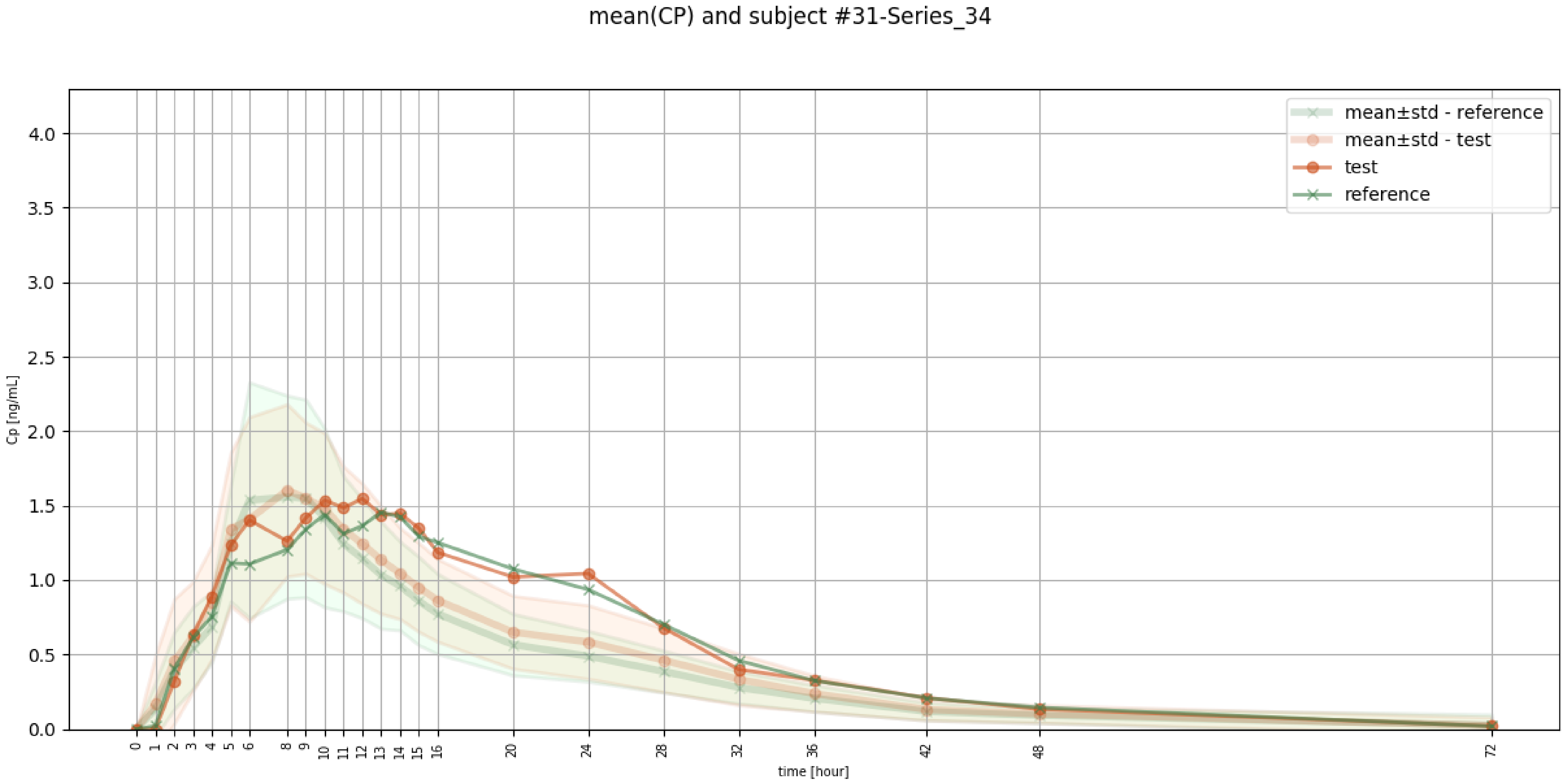

mean(CP) and subject #31-Series_34
mean±std - reference
mean±std - test
test
reference
Cp [ng/mL]
time [hour]
0.0
0.5
1.0
1.5
2.0
2.5
3.0
3.5
4.0
0 1 2 3 4 5 6 8 9 10 11 12 13 14 15 16 20 24 28 32 36 42 48 72

mean(CP) and subject #32-Series_34

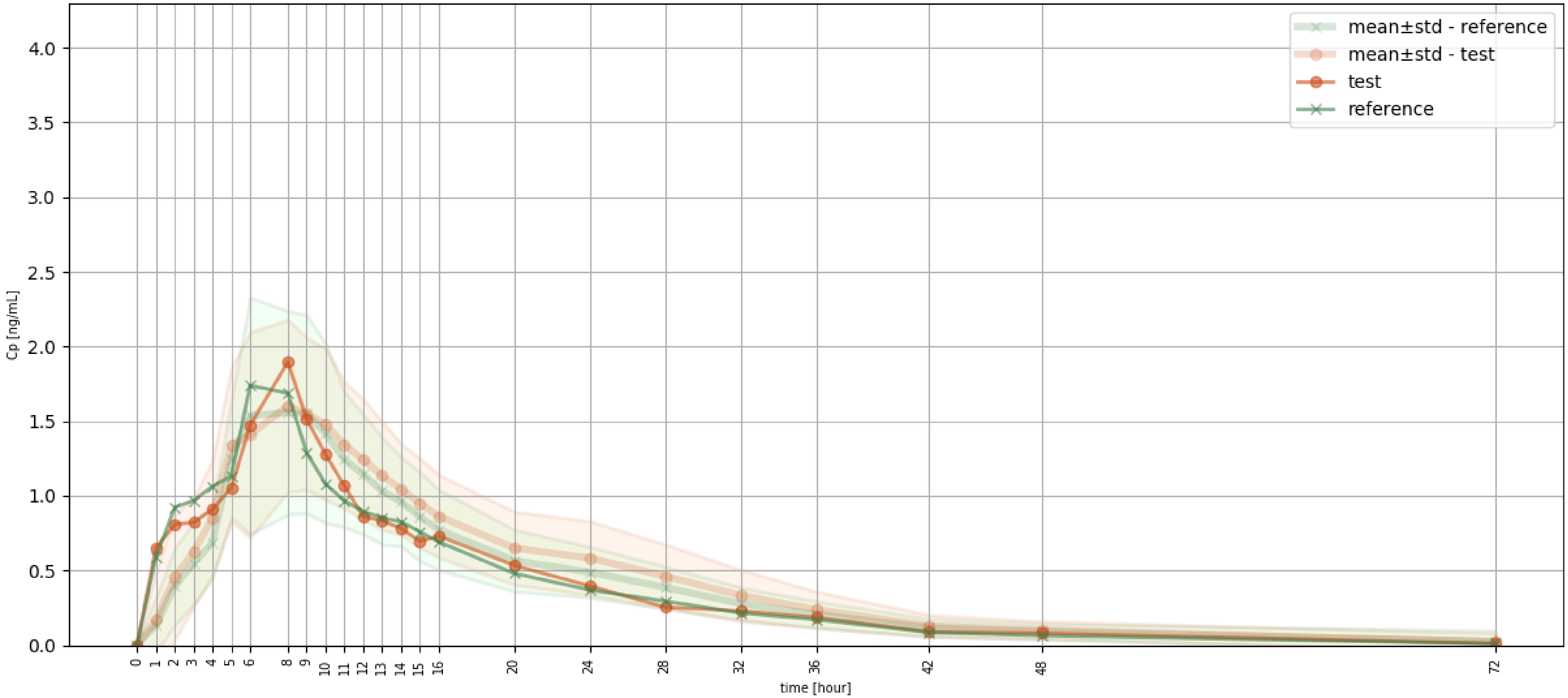

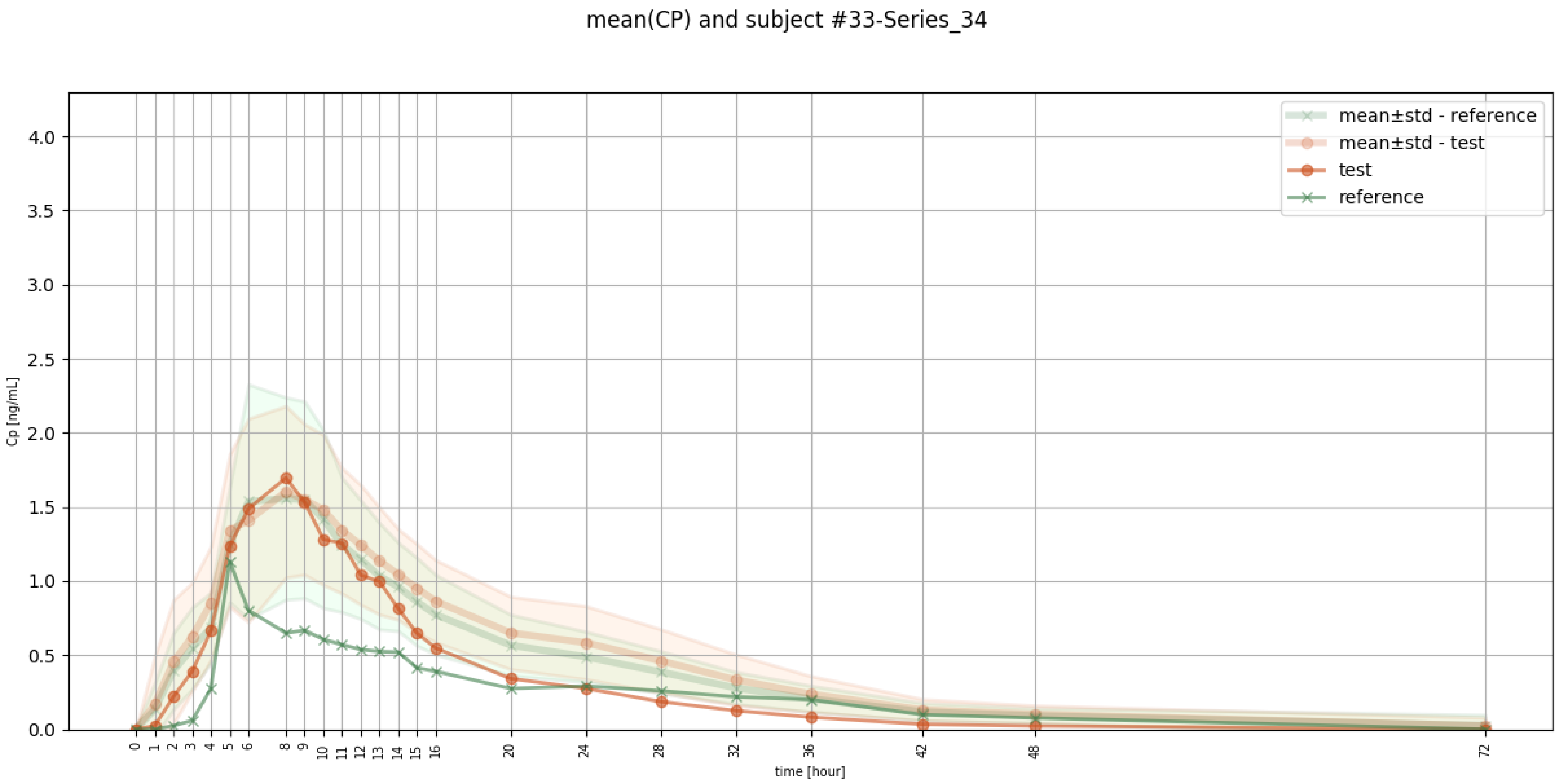
mean(CP) and subject #33-Series_34
mean±std - reference
mean±std - test
test
reference
Cp [ng/mL]
time [hour]
0.0
0.5
1.0
1.5
2.0
2.5
3.0
3.5
4.0
0
1
2
3
4
5
6
8
9
10
11
12
13
14
15
16
20
24
28
32
36
42
48
72

mean(CP) and subject #34-Series_34

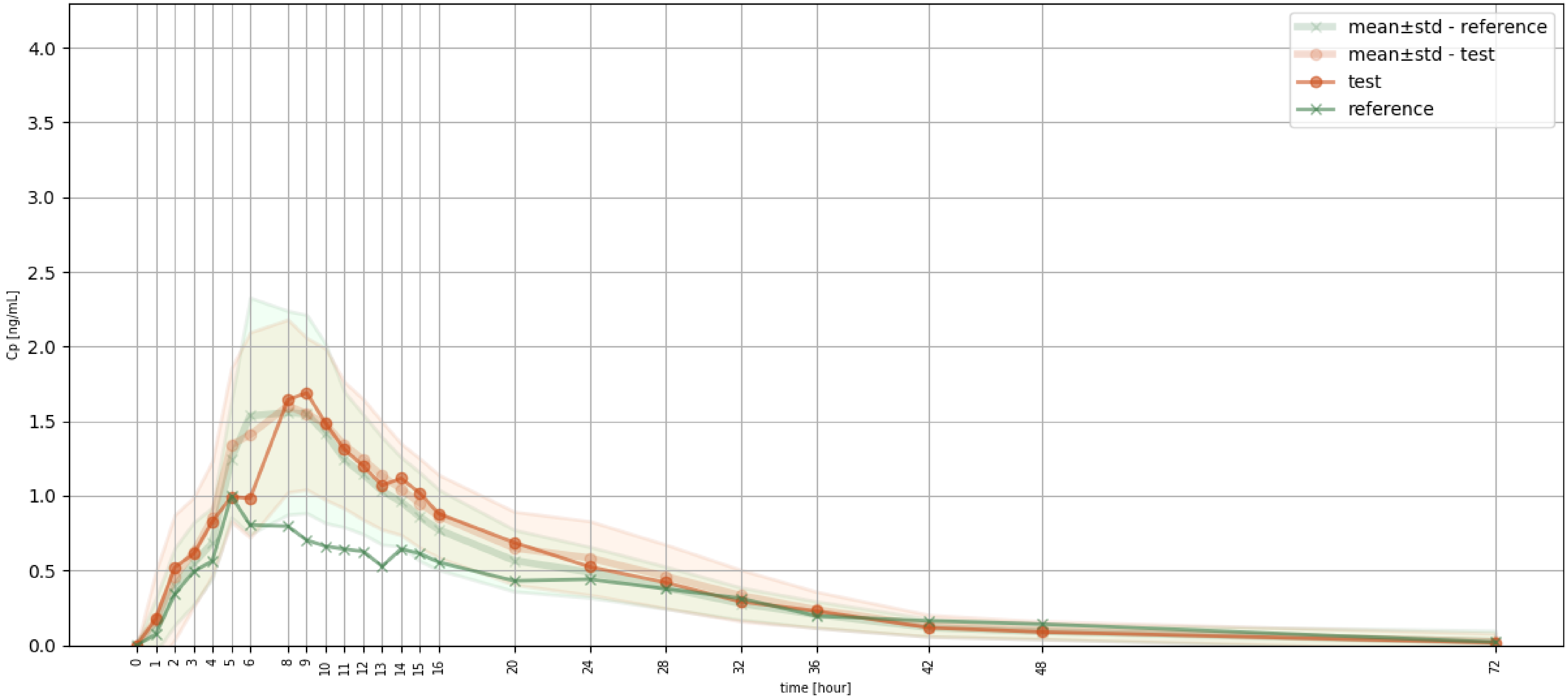

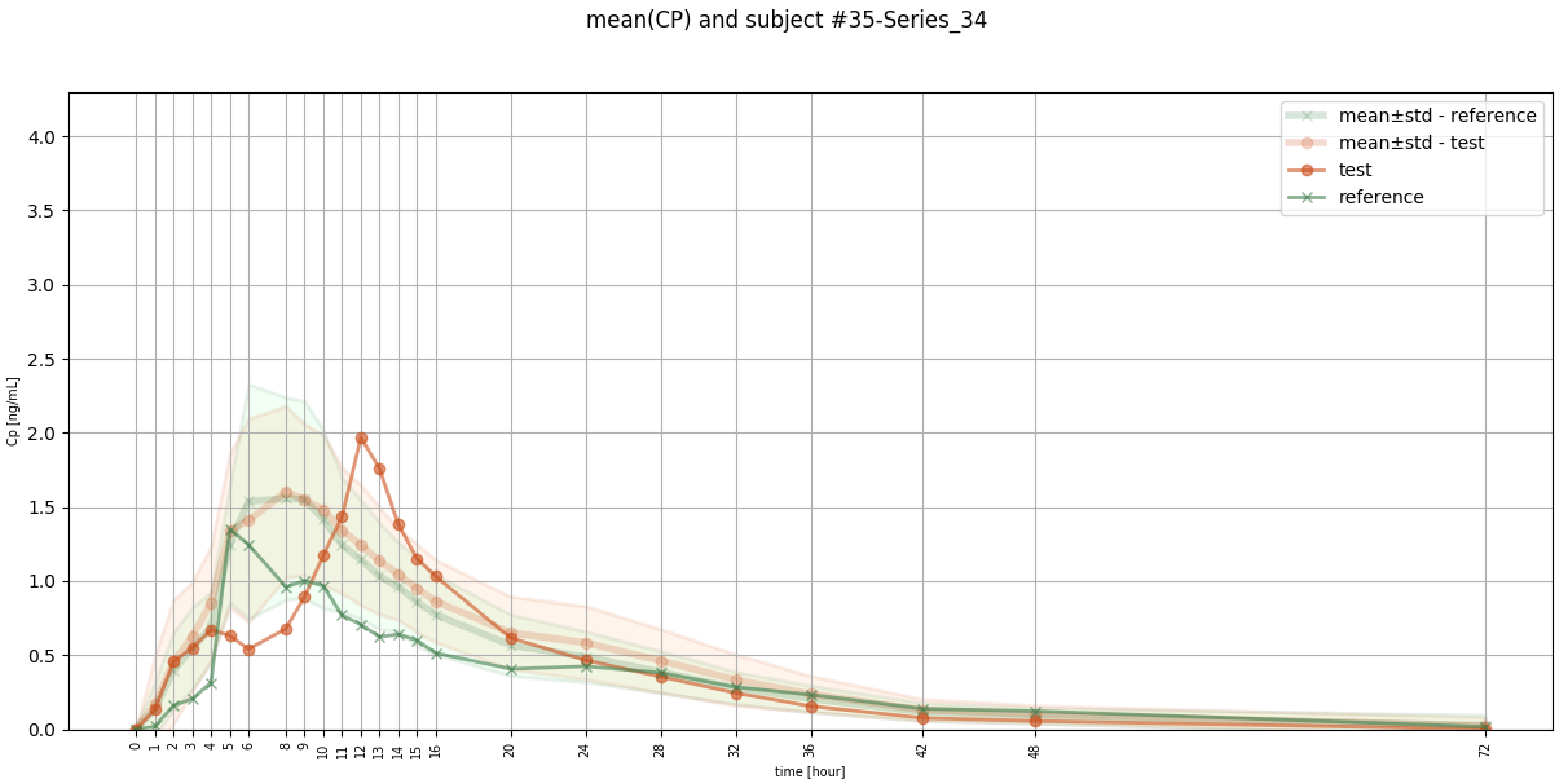
mean(CP) and subject #35-Series_34
mean±std - reference
mean±std - test
test
reference
Cp [ng/mL]
time [hour]
0.0
0.5
1.0
1.5
2.0
2.5
3.0
3.5
4.0
0 1 2 3 4 5 6 8 9 10 11 12 13 14 15 16 20 24 28 32 36 42 48 72

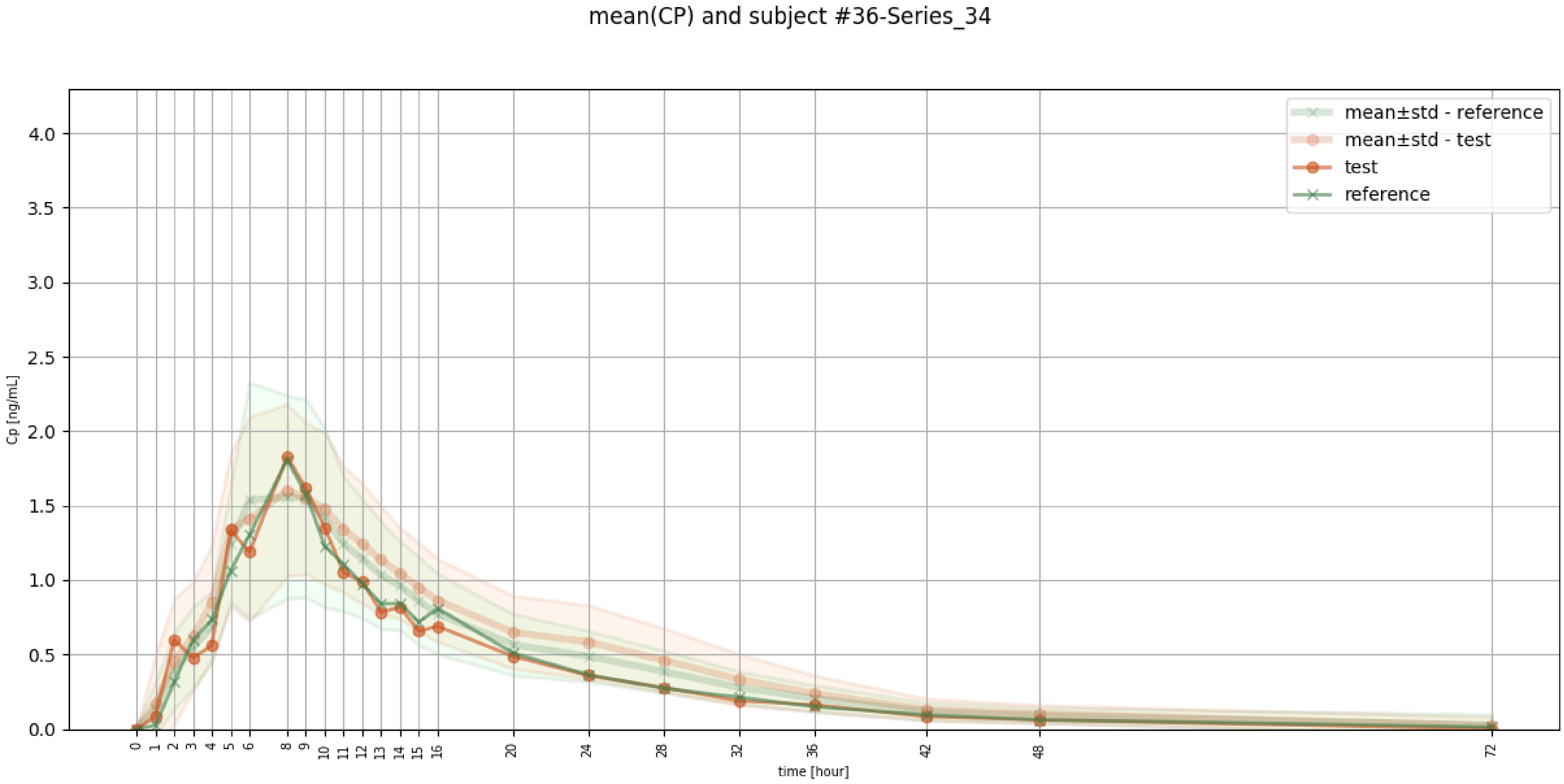
mean(CP) and subject #36-Series_34
mean±std - reference
mean±std - test
test
reference
Cp [ng/mL]
time [hour]
0.0
0.5
1.0
1.5
2.0
2.5
3.0
3.5
4.0
0 1 2 3 4 5 6 8 9 10 11 12 13 14 15 16 20 24 28 32 36 42 48 72

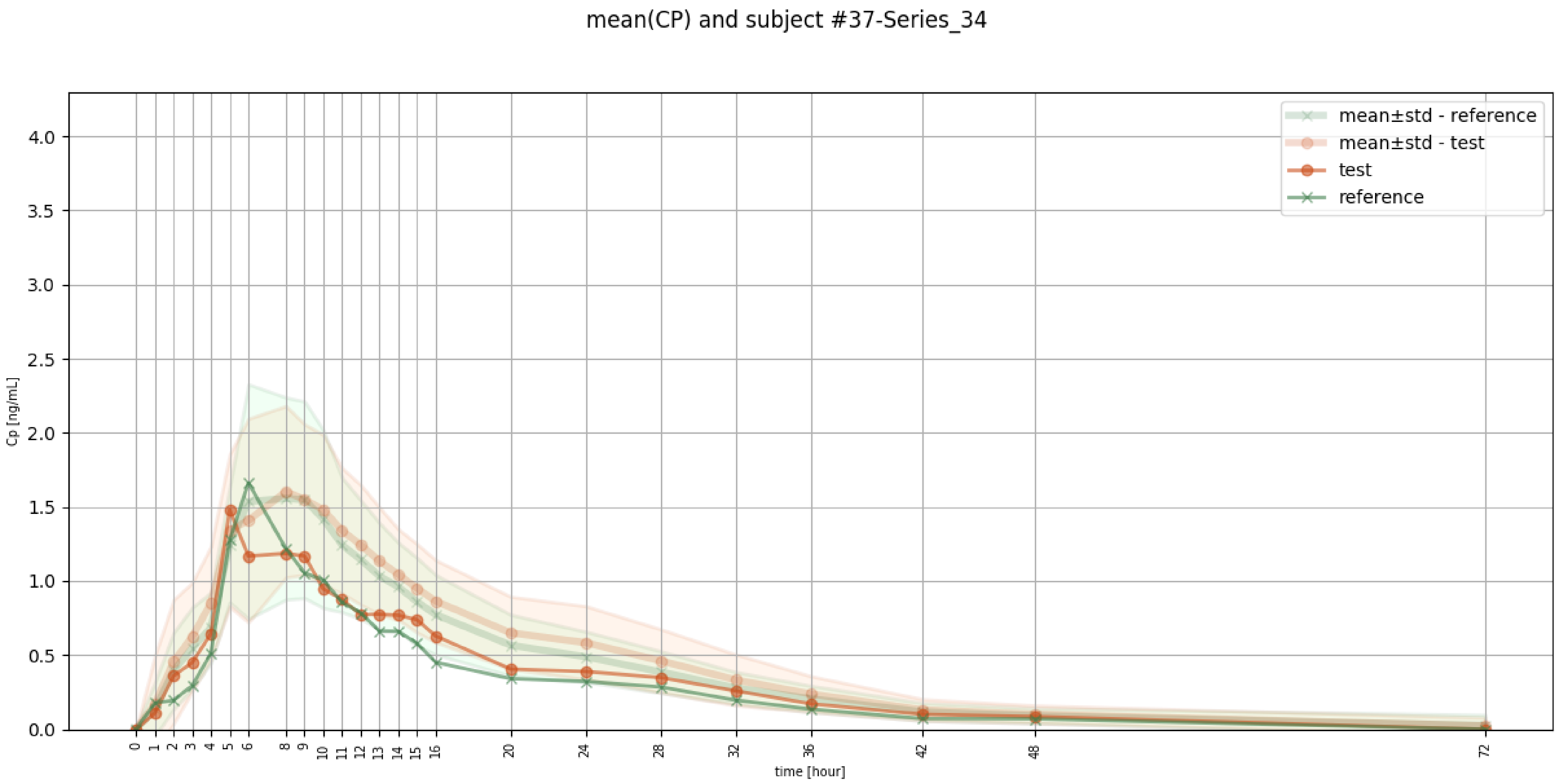

mean(CP) and subject #37-Series_34
mean±std - reference
mean±std - test
test
reference
Cp [ng/mL]
time [hour]
0.0
0.5
1.0
1.5
2.0
2.5
3.0
3.5
4.0
0 1 2 3 4 5 6 8 9 10 11 12 13 14 15 16 20 24 28 32 36 42 48 72

mean(CP) and subject #38-Series_34

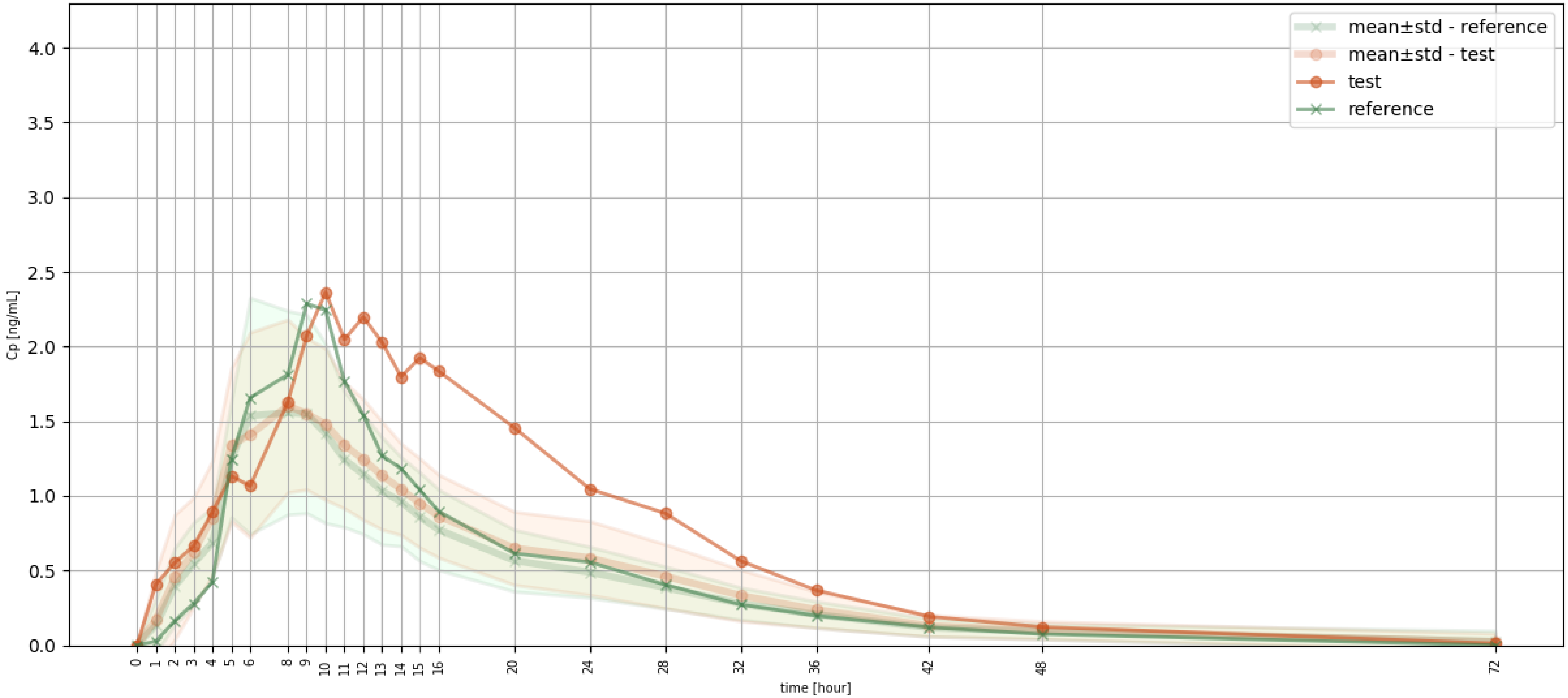

mean(CP) and subject #39-Series_34

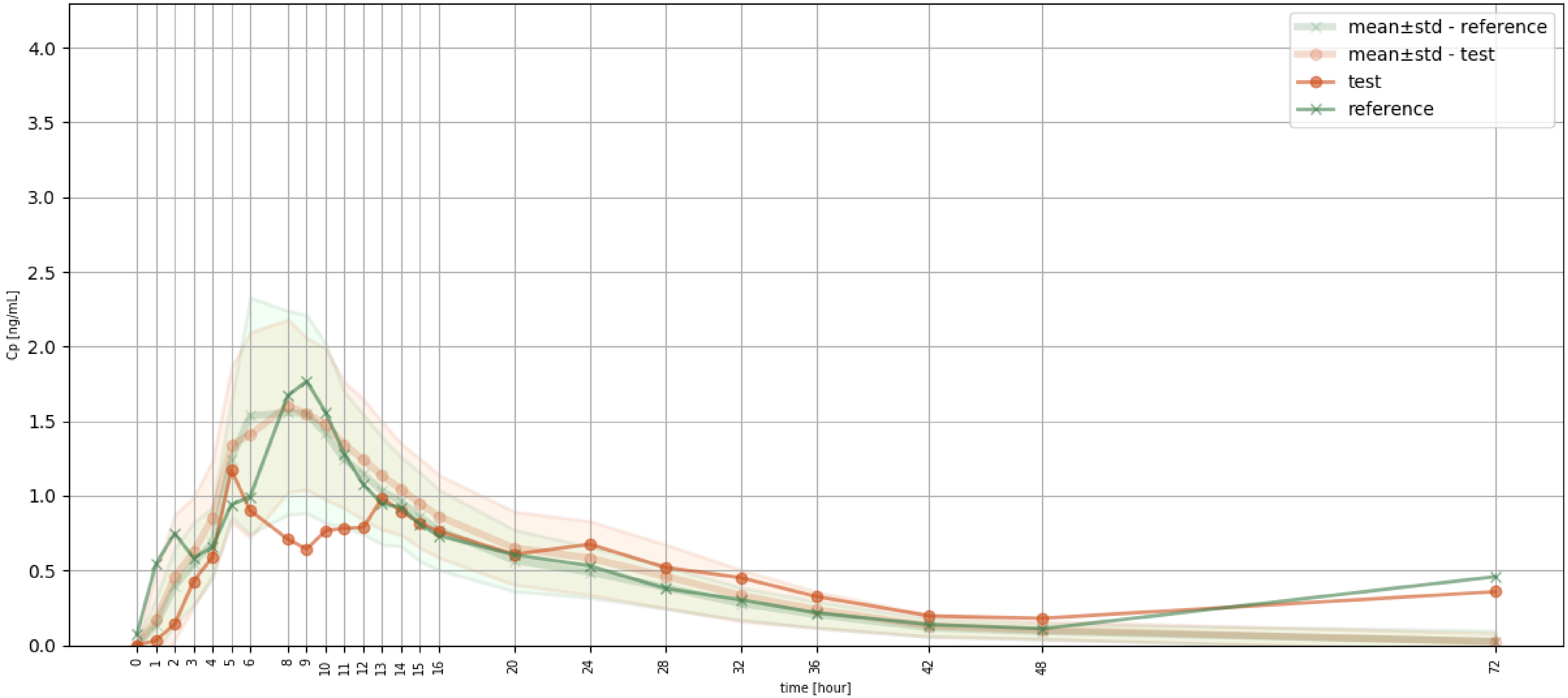

mean(CP) and subject #40-Series_34

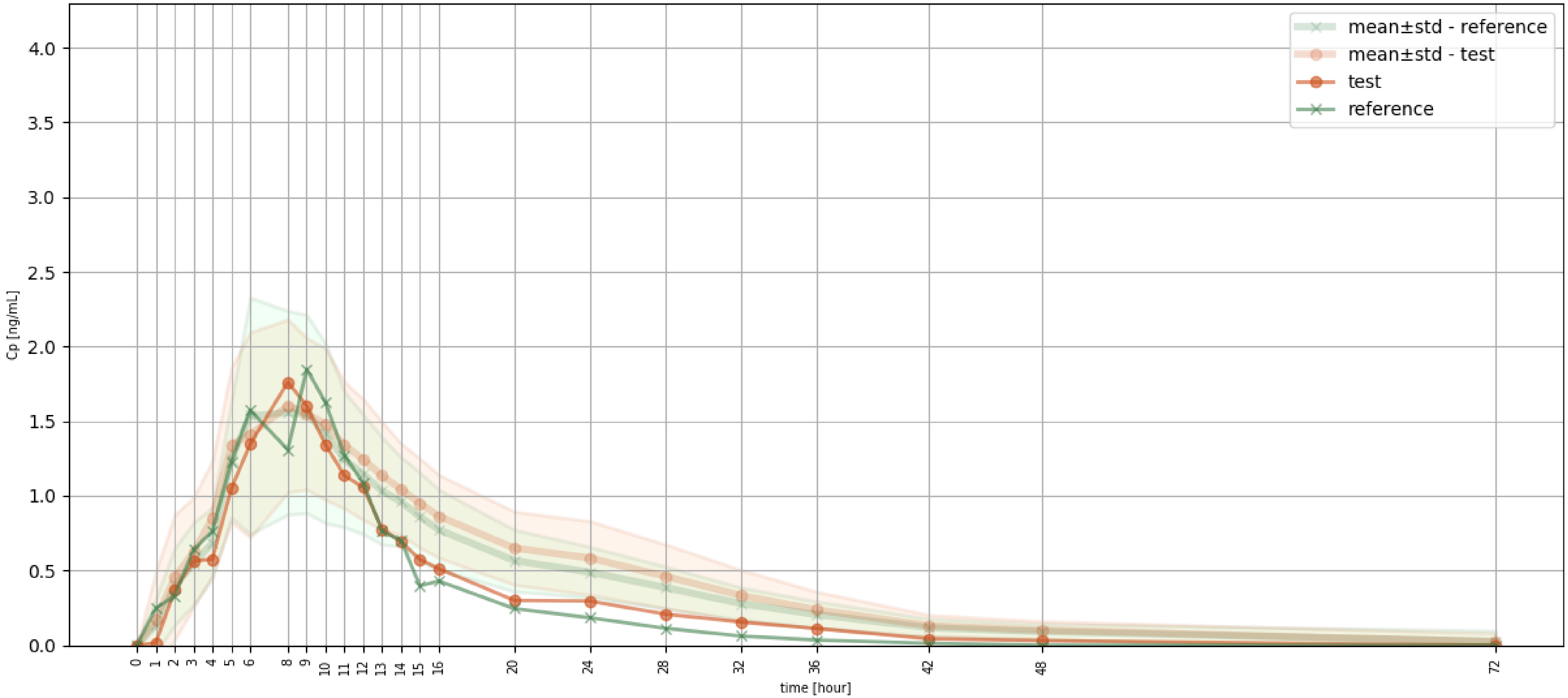

mean(CP) and subject #41-Series_34

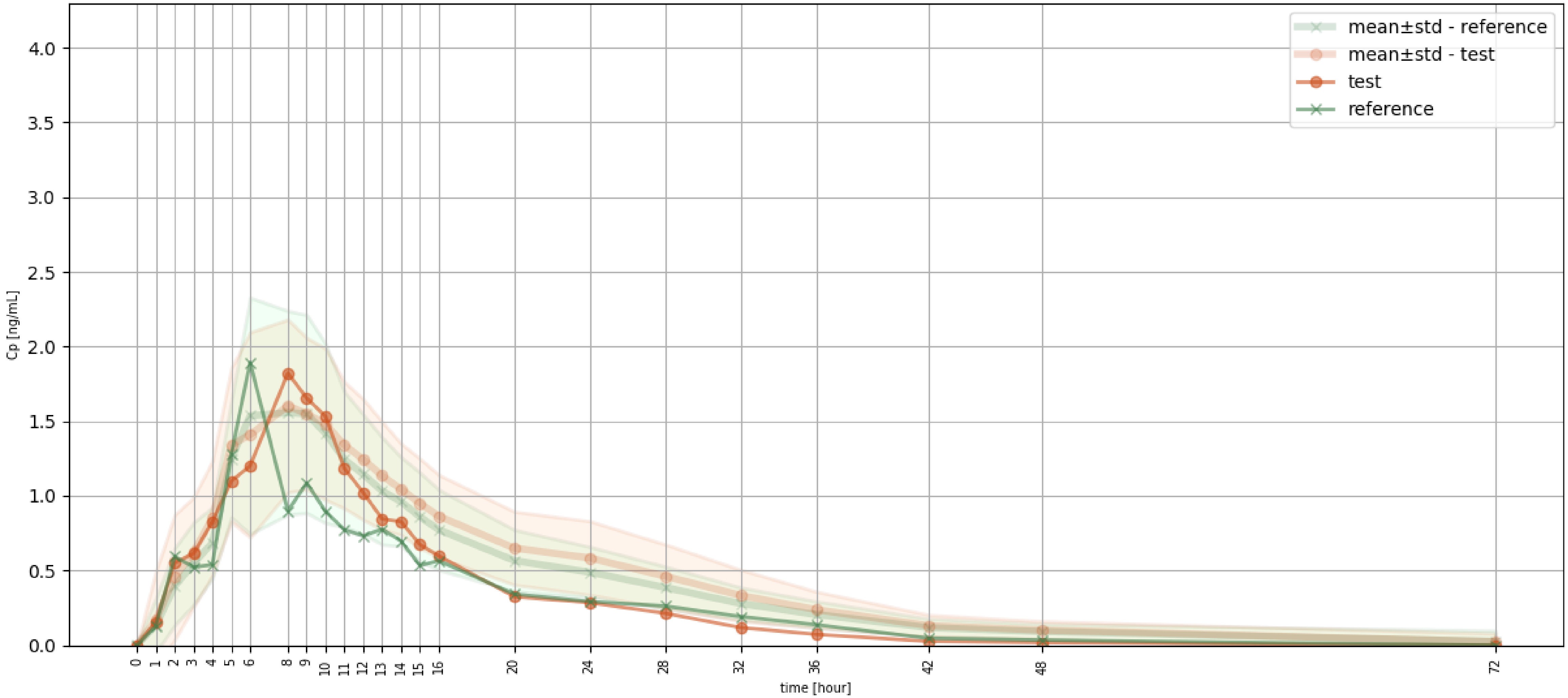

mean(CP) and subject #42-Series_34

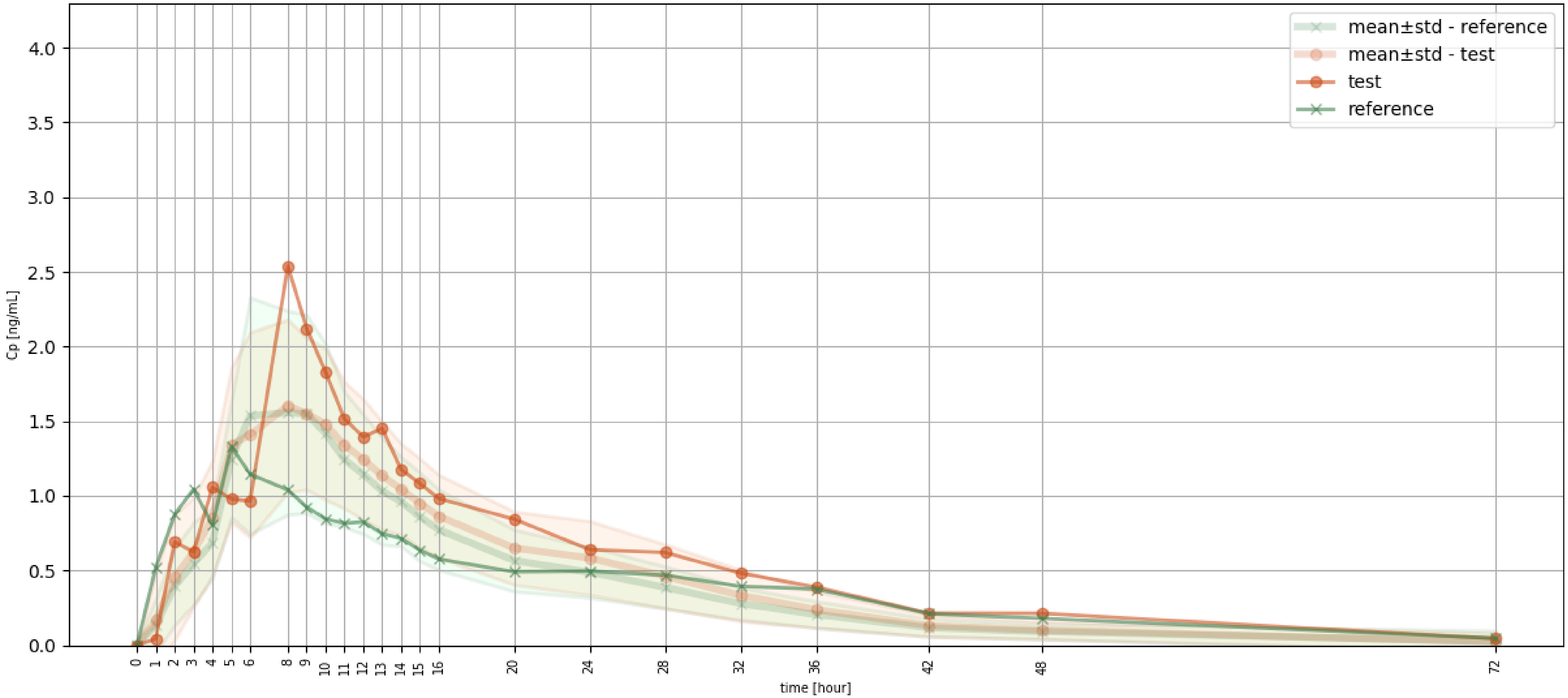

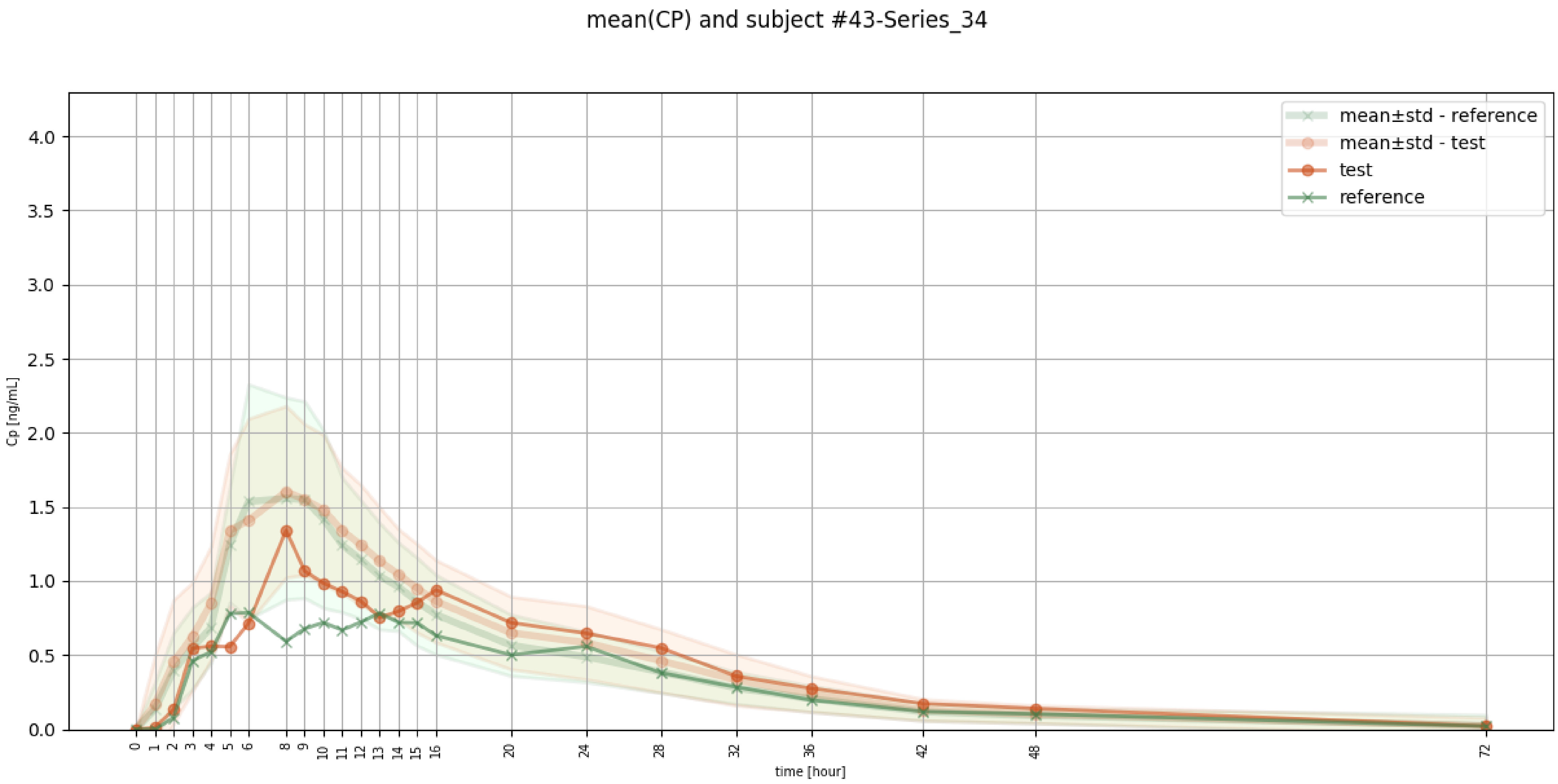

mean(CP) and subject #43-Series_34
mean±std - reference
mean±std - test
test
reference
Cp [ng/mL]
time [hour]
0.0
0.5
1.0
1.5
2.0
2.5
3.0
3.5
4.0
0
1
2
3
4
5
6
8
9
10
11
12
13
14
15
16
20
24
28
32
36
42
48
72

mean(CP) and subject #44-Series_34

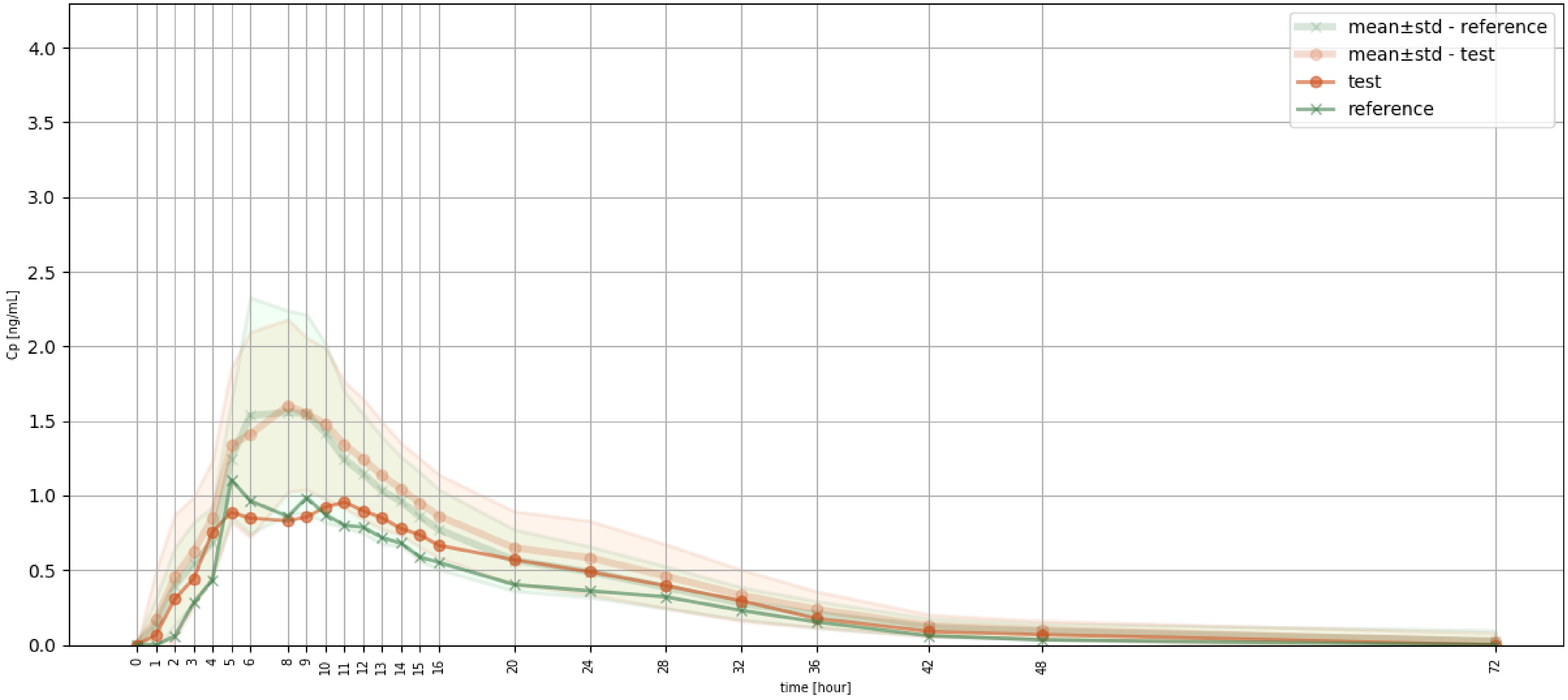

Supplement: Supplementary file 1 — (PDF 3410 kb) [file 12249_2020_1662_MOESM1_ESM.pdf]
